# Monitoring of Rice Transcriptional Responses to Contrasted Colonizing Patterns of Phytobeneficial *Burkholderia s.l.* Reveals a Temporal Shift in JA Systemic Response

**DOI:** 10.3389/fpls.2019.01141

**Published:** 2019-09-24

**Authors:** Eoghan King, Adrian Wallner, Isabelle Rimbault, Célia Barrachina, Agnieszka Klonowska, Lionel Moulin, Pierre Czernic

**Affiliations:** ^1^IRD, CIRAD, University of Montpellier, IPME, Montpellier, France; ^2^Montpellier GenomiX (MGX), c/o Institut de Génomique Fonctionnelle, Montpellier, France

**Keywords:** RNAseq, endophyte, symbiosis, *Burkholderia*, rice, jasmonic acid

## Abstract

In the context of plant–pathogen and plant–mutualist interactions, the underlying molecular bases associated with host colonization have been extensively studied. However, it is not the case for non-mutualistic beneficial interactions or associative symbiosis with plants. Particularly, little is known about the transcriptional regulations associated with the immune tolerance of plants towards beneficial microbes. In this context, the study of the *Burkholderia* rice model is very promising to describe the molecular mechanisms involved in associative symbiosis. Indeed, several species of the *Burkholderia sensu lato* (*s.l.*) genus can colonize rice tissues and have beneficial effects; particularly, two species have been thoroughly studied: *Burkholderia vietnamiensis* and *Paraburkholderia kururiensis*. This study aims to compare the interaction of these species with rice and especially to identify common or specific plant responses. Therefore, we analyzed root colonization of the rice cultivar Nipponbare using DsRed-tagged bacterial strains and produced the transcriptomes of both roots and leaves 7 days after root inoculation. This led us to the identification of a co-expression jasmonic acid (JA)-related network exhibiting opposite regulation in response to the two strains in the leaves of inoculated plants. We then monitored by quantitative polymerase chain reaction (qPCR) the expression of JA-related genes during time course colonization by each strain. Our results reveal a temporal shift in this JA systemic response, which can be related to different colonization strategies of both strains.

## Introduction

Plant microbiome is nowadays extensively studied, as it represents a huge potential for agriculture. Numerous studies describe the importance of microbes for plant’s nutrient supply and resistance to diseases and pests (Finkel et al., 2017). Therefore, microbes are potential solutions to do the transition to a sustainable agriculture while maintaining yield (Busby et al., 2017). Especially, some rhizobacteria have been shown to have tremendous beneficial effects on plant growth (Hayat et al., 2010) and resistance to pathogens (Beneduzi et al., 2012). These beneficial effects are induced through hormonal modulations following the colonization of plant roots (Vacheron et al., 2013) and inner tissues for endophytes (Hardoim et al., 2008) as well as systemic regulations of immunity (Pieterse et al., 2014). However, the perception of microbe-associated molecular patterns (MAMPs) generally leads to an immune response called MAMPs-triggered immunity (MTI) characterized by the synthesis of antimicrobial compounds (Jones and Dangl, 2006). In the same way pathogens suppress plant immunity, and beneficial microbes are able to escape (Trdá et al., 2015) or modulate (Zamioudis and Pieterse, 2012) the immune response of plants cells. Interestingly, the suppression of MTI by both pathogenic bacteria (Millet et al., 2010) and beneficial fungi (Jacobs et al., 2011) is commonly mediated *via* the jasmonic acid (JA) signaling pathway. However, the global physiological response and especially the transcriptional regulations induced by plants during the interaction with beneficial bacteria are not well described.

The interaction between plants and rhizospheric or endophytic bacteria-forming associative symbioses has been studied in several species belonging to the genera *Azoarcus*, *Azospirillum*, *Herbaspirillum*, *Acetobacter*, *Gluconacetobacter*, *Bacillus*, *Phyllobacterium*, *Pseudomonas*, and (*Para*-)*Burkholderia* (Ahemad and Kibret, 2014). Most studies on these models focused on the bacterial response to the interaction with its host plant (Shidore et al., 2012; Coutinho et al., 2015; Sheibani-Tezerji et al., 2015), while relatively few studies analyzed the transcriptional response of plants. Nonetheless, several studies described plants’ transcriptional regulations including lowering of defense (Bordiec et al., 2011; Rekha et al., 2018), hormonal signaling (Drogue et al., 2014; Rekha et al., 2018), developmental reprogramming (Paungfoo-Lonhienne et al., 2016), and iron homeostasis (Brusamarello-Santos et al., 2019; Stringlis et al., 2018).

Within the diversity of bacteria interacting with plants, the *Burkholderia sensu lato* (*s.l.*) genus of Betaproteobacteria stands out for several reasons. It contains plant pathogenic species (*B glumae*, *Burkholderia gladioli*, and *Burkholderia plantarii*) (Maeda et al., 2006), N_2_-fixing nodule-forming species in association with tropical legumes (Gyaneshwar et al., 2011) as well as species-forming associative symbiosis particularly with cereals such as rice (Coutinho et al., 2013; Govindarajan et al., 2008). Also, a phylogenetic separation discriminates the *Burkholderia sensu stricto* (*s.s.*) genus, which contains animal or plants pathogens as well as human opportunists, and the *Paraburkholderia* genus, which contains mainly plant-associated and environmental species (Sawana et al., 2014; Estrada-de los Santos et al., 2016). Also, most recent phylogenetic refinements of *Burkholderia s.l.* taxa defined other genera—*Caballeronia*, *Robbsia*, *Trinickia*, and *Mycetohabitans*—which are supported by both differences in genomic and ecological features (Estrada-de los Santos et al., 2018).

Interestingly, two strains of the *Burkholderia s.l.* genus able to fix N_2_ have been described as growth promoters in rice. *Paraburkholderia kururiensis* M130 (hereafter *Pk*) is a beneficial rice endophyte (Mattos et al., 2008) related to environmental and plant-beneficial strains (Kaur et al., 2017). *Burkholderia vietnamiensis* TVV75^T^ (hereafter *Bv*) is a rice-associated species having positive effect on yield (Trân Van et al., 2000; Govindarajan et al., 2008), which belongs to the *Burkholderia cepacia* complex, a complex of species that can cause serious risks to cystic fibrosis patients (Vial et al., 2011). In order to decipher how rice perceives these two beneficial strains belonging to genera with contrasted ecologic backgrounds, we studied the transcriptional responses of rice during the establishment of the interaction. Our aim was to identify plant physiological processes and potentially key genes involved in beneficial rice-rhizobacteria interactions and also differentially regulated by each strain. We first analyzed the colonization patterns of *Pk* and *Bv* on the *Oryza sativa* Nipponbare genotype. We then analyzed the root and leaf transcriptional responses to the bacterial colonization by RNAseq. This led us to the identification of a co-expression JA-related network. Therefore, we monitored, throughout the establishment of the interaction, the expression of JA-related genes by reverse transcriptase–quantitative polymerase chain reaction (RT-qPCR).

## Materials and Methods

### Plants and Bacterial Cultivation

*O. sativa* L. ssp. *japonica* cv Nipponbare was used in this study. For all experiments, seeds were dehusked and sterilized as follows: 70% ethanol for 10 min and 9.6% NaClO supplemented with 1% Tween 20 for 30 min. Treated seeds were rinsed twice with sterile distilled water, twice with 2% thiosulfate solution, and finally four times with sterile distilled water. To confirm surface sterilization, 100 µl of the last rinsing solution was plated in tryptic soy agar (TSA) medium (Sigma-Aldrich). Seeds were then put in sterile distilled water at 28°C for 24 h and transferred on 8% H_2_O agar plate for 30 h. Homogeneously germinated seeds were transferred to sterile magenta boxes (SPL Lifesciences Co. Ltd) containing 150 ml of autoclaved perlite and 200 ml of sterile hydroponic medium (recipe in **Supplementary Table 1**). Plants were grown in a growth chamber (16 h light; 8 h dark; 28°C; 70% humidity).

All bacterial strains (listed in **Supplementary Table 2**) were cultured as follows: Glycerol stocks (20%) of bacterial cells conserved at −80°C were plated in low-salt lysogeny broth (LB) (Sigma-Aldrich) agar plates and incubated for 72 h at 28°C. Liquid low salt LB medium of 20 ml was then inoculated in 50-ml Falcon tubes and incubated for 16 h under agitation (180 rpm) at 28°C. Overnight culture of 500 µl was inoculated in fresh liquid medium for 2 h. Bacterial cells were then centrifuged for 5 min at 4,000 rpm and resuspended in sterile distilled water. Each plantlet was inoculated with 10^7^ bacterial cells 4 days after sowing in hydroponic system.

### Bacterial Transformation

*P. kururiensis* M130 and *B. vietnamiensis* TVV75 cells were transformed by electroporation with the pIN29 plasmid (Vergunst et al., 2010). The plasmid chosen to transform the strains, pIN29, comprises a chloramphenicol resistance gene as well as the DsRed gene under the control of a constitutive TAC promoter. After 24 h of incubation of selective medium low salt LB Cm (200 μg·ml^−1^) at 28°C, the most fluorescent colonies were selected.

### Rice Root Colonization Assays

The roots of plants were harvested at 1, 7, and 14 days postinoculation (dpi), weighted, and grinded in sterile water with a sterile ceramic bead using a FastPrep-24^™^ 5G at 6 m·s^−1^ for 40 s. The solution was then diluted and inoculated in low-salt LB selective medium containing 200 μg·ml^−1^ of chloramphenicol and incubated at 28°C for 24 h. Colony-forming units were then enumerated. The size of the root-associated bacterial population was measured during two independent experiments, each comprising nine plants. In order to measure the size of the endophytic population, the inoculated rice roots were surface disinfected for 1 min using a solution of 1% chloramine T (Sigma-Aldrich) supplemented with 0.1% Tween 20. Roots were then rinsed six times with sterile water. Controls of disinfection were performed by plating rinsing water in TSA medium (Sigma-Aldrich) overnight. Surface-disinfected roots were then treated as previously described. The size of the endophytic population was measured on five plants.

### Microscopy

All microscopic observations of the bacterial colonization were restricted to the primary root in order to compare the colonization patterns on roots that have been in contact with the bacterial population for the same amount of time. Primary roots were harvested at 7 and 14 dpi and mounted between slide and slips cover and directly examined with the microscopes. Epifluorescence observations were performed using a Nikon Eclipse Ni-E microscope. Confocal Laser Scanning observations were performed using a Zeiss LSM880 confocal microscope.

### RNA Extraction

For the analysis of root and leaf transcriptional profiles, both plants’ tissues were harvested at 6 h postinoculation (hpi), 1 dpi, 7 dpi, or 14 dpi with live bacterial cells. Each biological replicate consisted of five pooled root system or five pooled last mature leaves harvested from a single hydroponic system. For each time point and each inoculated strain, three biological replicates were harvested. Roots and leaves of untreated plants were collected at the same time points. After harvest, samples were snap-frozen in liquid nitrogen and stored at −80°C.

Rice roots were homogenized in liquid nitrogen using cooled mortar and pestle. Rice leaves were grinded using a TissueLyser II (Retsch) set to 30 Hz for 30 s. Total RNA extraction using TRI-reagent (Sigma) was performed according to manufacturer’s instructions. All samples were treated with DNase I (Ambion) and purified using the RNA Clean & Concentrator kit (Zymo) according to manufacturer’s instructions. The integrity and quality of the total RNA were confirmed using a NanoDrop^™^ 1000 spectrophotometer (Thermo Fisher) and a 2100 BioAnalyzer (Agilent).

### RNA Sequencing and Mapping of Reads

Quality of RNA was checked by determining the RNA Integrity Number (RIN) with a Fragment Analyzer (Agilent). For the library preparation, samples with a RIN value > 6 were used. Eighteen RNA libraries were prepared using an Illumina TruSeq stranded mRNA sample preparation kit by MGX-Montpellier GenomiX core facility (MGX) France (https://www.mgx.cnrs.fr/). Library construction and sequencing were performed as described in Karmakar et al. (2019) on an Illumina HiSeq 2500. The quantitative and qualitative validation of the library was performed by qPCR, Roche LightCycler 480, and a Fragment Analyzer (Agilent) using a Standard Sensitivity NGS kit. Quality control and assessment of raw Illumina reads in FASTQ format were done by FastQC software (version 0.11.5) to obtain per base quality, Guanine-Cytosine (GC) content, and sequence length distribution. Clean reads were obtained by removing the low-quality reads, adapters, and poly-N-containing reads by using Trimmomatic v0.36 software (Bolger et al., 2014). RNAseq reads were aligned to the IRGSP 1.0 version of the rice genome using HISAT2 v2.0.5.1 (Kim et al., 2015). The number of reads mapped to each gene locus was counted using HTSEq-count v0.6.0 (Anders et al., 2015).

### Differential Gene Expression and Gene Ontology (GO) Term Enrichment Analysis

DESeq2 v3.7 (Love et al., 2014) was used to calculate differential gene expression between non-inoculated and inoculated conditions. All genes having an adjusted *p*-value inferior to 0.01 were considered as significantly differentially expressed. All functional enrichment analyses were performed using g:Profiler (version e95_eg42_p13_f6e58b9) with g:SCS multiple testing correction method applying significance threshold of 0.05 (Reimand et al., 2007; Raudvere et al., 2019).

### Quantification of mRNA Levels Using RT-qCPR

cDNA was produced from 350 ng of DNase-treated total RNA using the SuperScript III Reverse Transcriptase (Thermo Fisher Scientific). The cDNA reaction was diluted five times before qPCR using MESA BLUE qPCR Master Mix for SYBR^®^ assay (Eurogentec) on an Mx3005P qPCR system (Agilent Technologies). The relative expression level was calculated according to Pfaffl (2001). Three independent samples were analyzed for each condition, and each sample was assayed in triplicate. Primers used are listed in **Supplementary Table 3**.

## Results

### Analysis of Root Colonization

**Figure 1:**
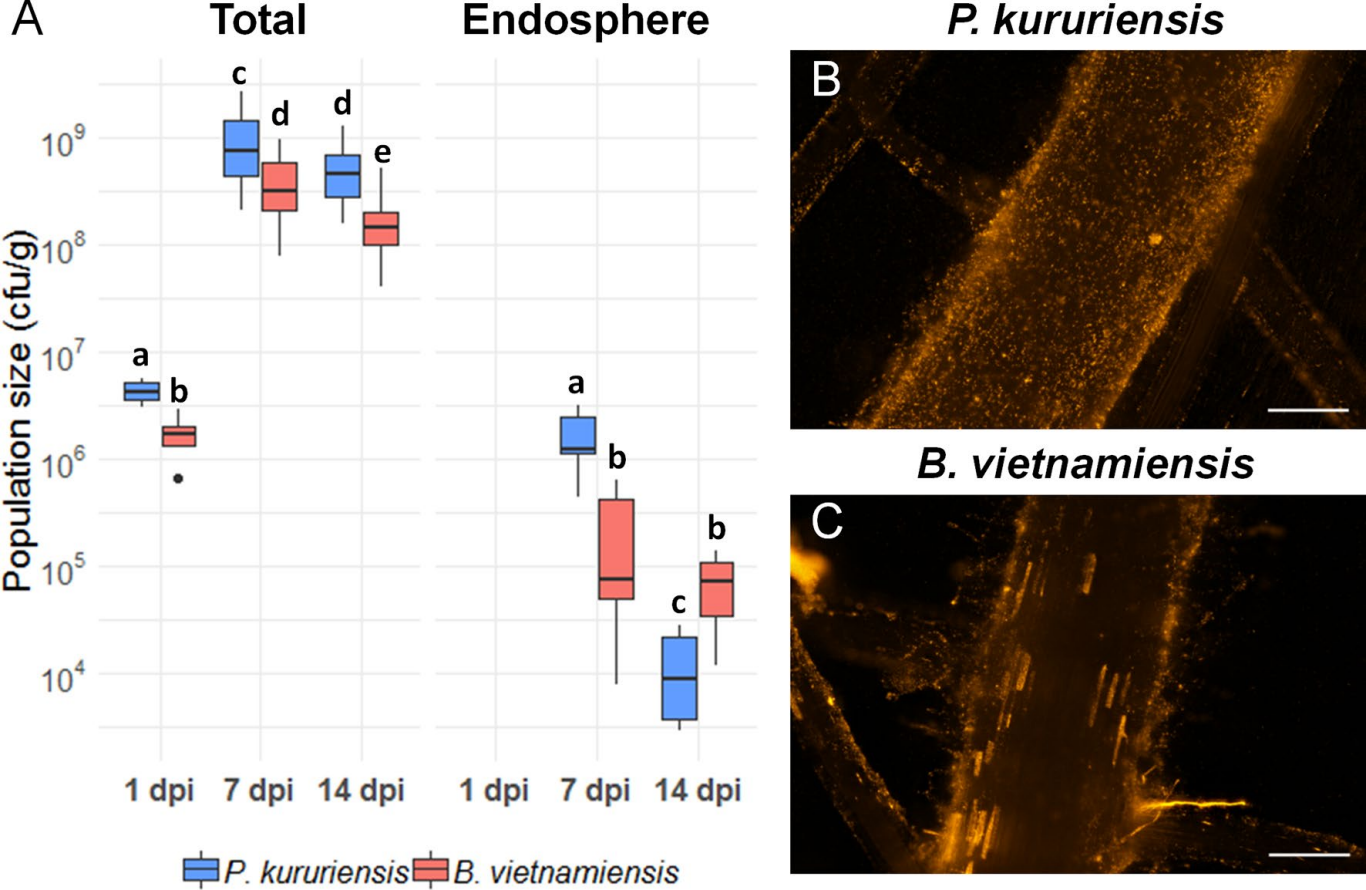
Colonization of the roots of hydroponically grown rice plants by *Bv* and *Pk*. **(A)** Population dynamics of DsRed-tagged *Bv* and *Pk* associated with rice roots and inside the plant roots. The data reported are the median of bacterial population size from 18 plants and two independent experiments for rhizosphere compartment and five plants for the endophytic compartment. The letters indicate the significance groups in each compartment according to *post-hoc* tests on a generalized linear model. Epifluorescence microscopy pictures of the colonization of rice primary roots at 7 days postinoculation by *Pk* **(B)** and *Bv* **(C)** DsRed cells. White bars represent 200 µm. *Bv*, *Burkholderia vietnamiensis* TVV75^T^; *Pk*, *Paraburkholderia kururiensis* M130.

We used hydroponic culture of rice plants, grown in axenic condition and inoculated with DsRed-tagged strains, to monitor the bacterial colonization at 1, 7, and 14 dpi. First, the root’s colonization was measured by counting the bacterial populations on the rhizoplan and endosphere (see Materials and Methods). The roots of rice plants were rapidly colonized by both bacterial strains (**Figure 1A**). The populations of *Pk* and *Bv* reach a median value of 4.16 × 10^6^ and 1.73 × 10^6^ cfu·g^−1^, respectively, at 1 dpi. At 7 dpi, the maximum measured size of the bacterial population is reached for both strains: 7.49 × 10^8^ and 3.18 × 10^8^ cfu·g^−1^ for *Pk* and *Bv*, respectively. Then, between 7 and 14 dpi, the size of the total root-associated population decreases for each strain, reaching a median value of 4.6 × 10^8^ and 1.46 × 10^8^ cfu·g^−1^ for *Pk* and *Bv*, respectively. Between the same time points, the variation of the endophytic population size differs between the two strains. Indeed, the endophytic population size of *Pk* decreases from 1.25 × 10^6^ to 1.17 × 10^4^ cfu·g^−1^ between 7 and 14 dpi, while the median number of endophytic *Bv* cells remain stable with 7.34 × 10^4^ and 7.5 × 10^4^ cfu·g^−1^ at 7 and 14 dpi, respectively.

**Figure 2:**
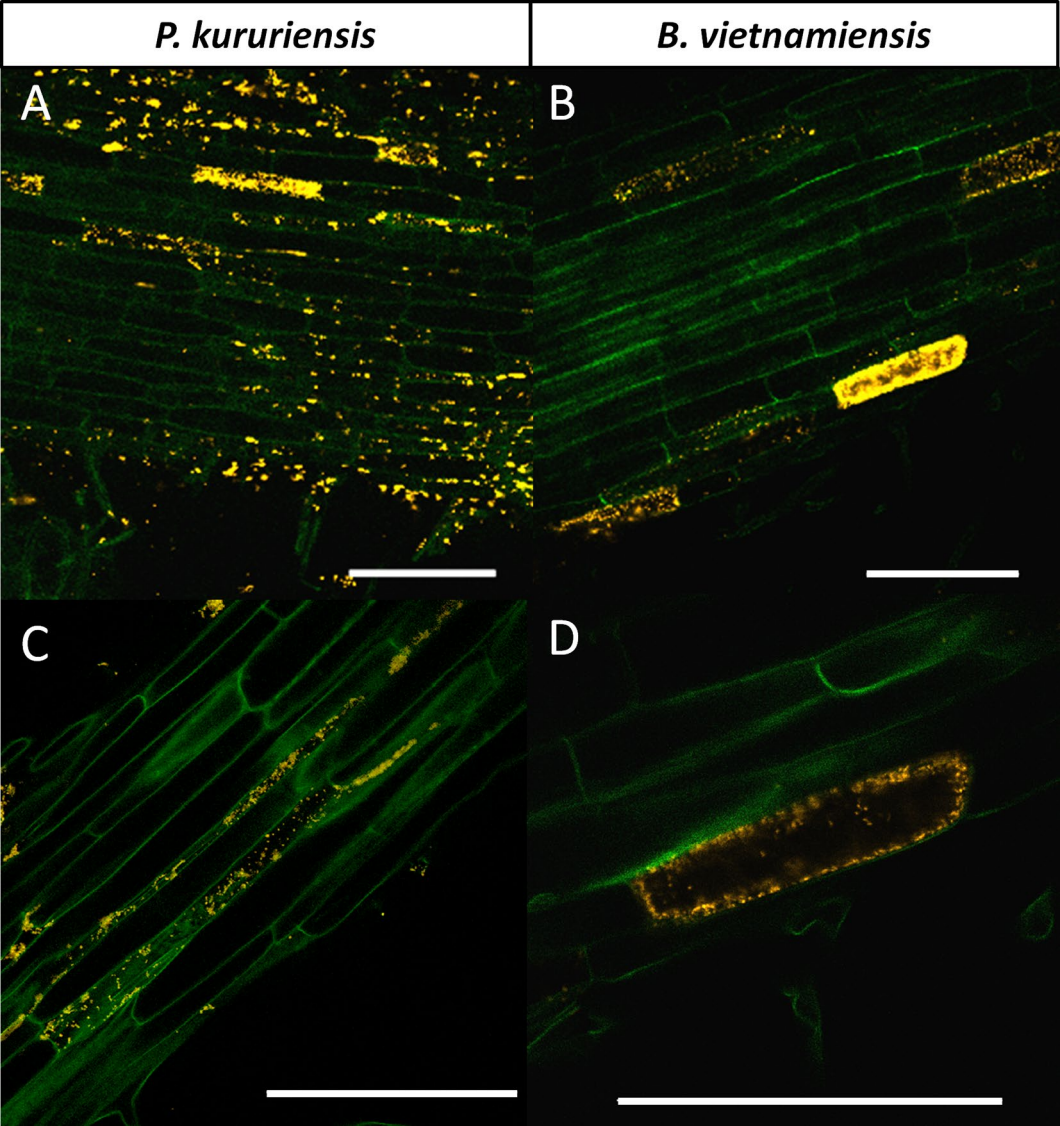
Endophytic colonization of the roots of hydroponically grown rice plants by *Bv* and *Pk*. Confocal microscopy observations of *Pk* and *Bv* DsRed-tagged cells colonizing the inner tissues of rice roots at 14 days postinoculation. **(A)** *Pk* cells colonizing the surface and the intercellular spaces of epidermic cells. **(B)** Multiple epidermic cells colonized by *Bv* cells. **(C)** Apoplastic colonization by *Pk* cells. **(D)** Rice root epidermic intracellular colonization by *Bv* cells. White bars represent 100 µm. *Bv*, *Burkholderia vietnamiensis* TVV75^T^; *Pk*, *Paraburkholderia kururiensis* M130.

Microscopic observations of the primary roots of inoculated rice plants demonstrated that both bacterial strains tagged with the DsRed gene colonized the root surface after inoculation (**Figures 1B, C**). Moreover specific zones were more densely colonized by bacteria such as the surface of root hairs and the emergence of lateral roots (**Supplementary Figure 1**). By comparing the colonization of the two strains, differences can be observed in the way they colonized the surface of the primary root. Several epidermal plant cells seemed colonized intracellularly by the tagged *Bv* cells, while the phenomenon was observed less frequently in *Pk*-colonized roots (**Figure 1C**; **Supplementary Figure 1**). Observations by confocal microscopy revealed that for *Pk*, the highly colonized epidermal cells appear to be only colonized on their surface as the whole outline of epidermal cells as well as in intercellular spaces (**Figures 2A, C**), while for *Bv*, most of the observations show that bacterial cells were able to cross the cell wall and were observed in the cytoplasm of the cell (**Figures 2B, D**). Thus, both strains colonized the roots of the Nipponbare cultivar both externally and endophytically but through apparently different intensities and entry roads. We then wondered if the host plant induces contrasted transcriptional regulations associated with the differential colonization pattern of each strain.

### Transcriptional Response of Rice to Bacterial Inoculation

In order to identify changes in *O. sativa* transcriptome in response to both strains, we performed RNAseq on leaves and roots of non-inoculated controls, *Pk*-inoculated and *Bv*-inoculated plants at 7 dpi. We chose this time point to avoid the initial plant defense burst due to a bacterial inoculation in hydroponic system (hours to 1 dpi) and allow an advanced colonization stage of the roots but without any visible developmental effect such as increased growth. To confirm that the inoculated and non-inoculated control plants were at the same developmental stage, we measured the dry weight of the plants and could not detect any significant impact of the inoculation on biomass production (**Supplementary Figure 2**) nor on plant height. Therefore, we assume that the differences between the transcriptomes of inoculated plants and non-inoculated controls should be related to bacterial colonization of the roots rather than a developmental impact of inoculation.

**Figure 3:**
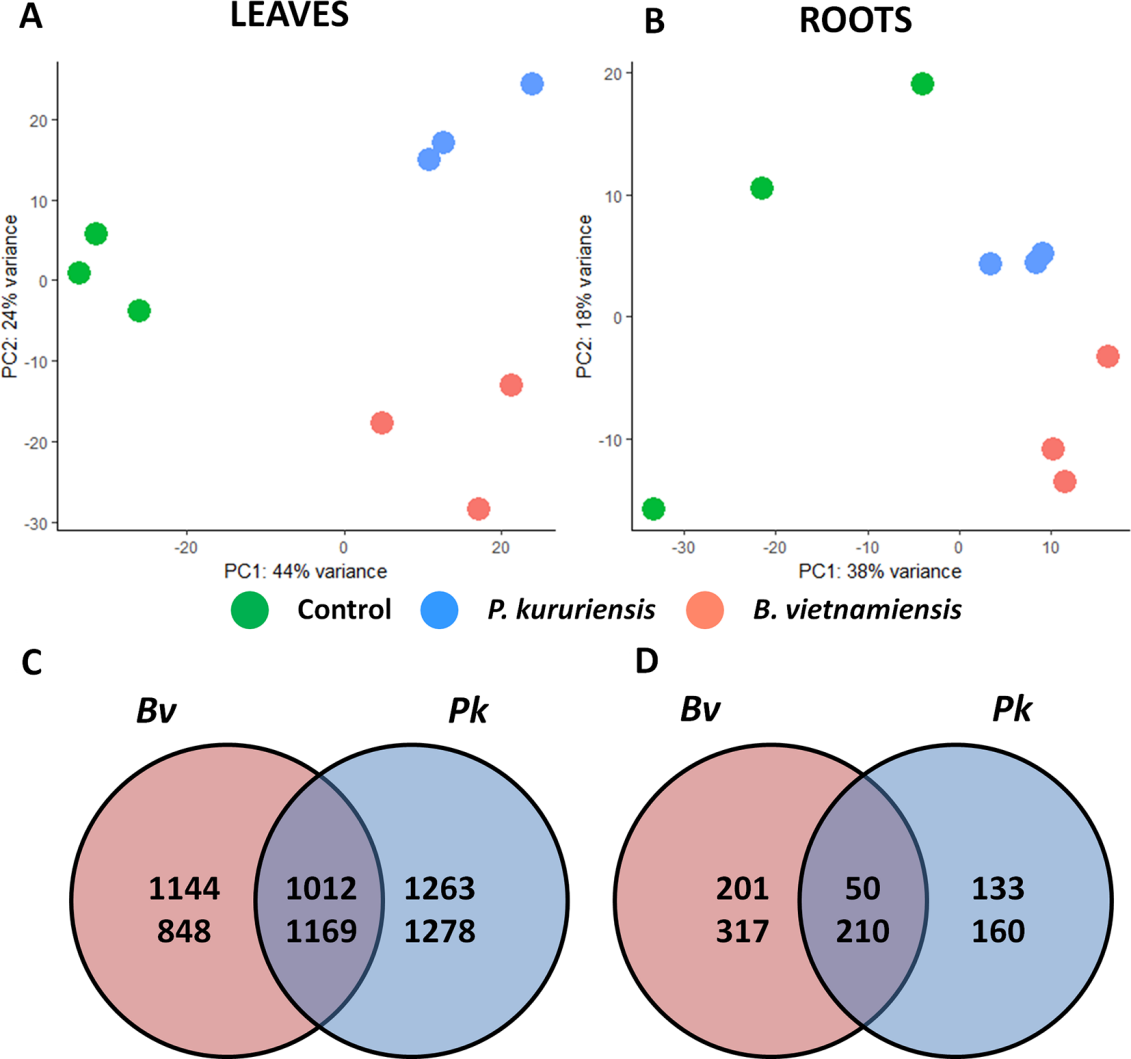
Comparative analysis of leaves and roots transcriptomes in response to *Bv* and *Pk* colonization. Principal component analysis of the normalized number of reads mapped per gene in leaves **(A)** and in roots **(B)**. Number of genes regulated in leaves **(C)** and in roots **(D)** following bacterial inoculation, FDR < 0.01. The numbers on the upper part correspond to the number of genes up-regulated, inversely for the number of down-regulated genes. *Bv*, *Burkholderia vietnamiensis* TVV75^T^; *Pk*, *Paraburkholderia kururiensis* M130; FDR, false discovery rate.

A total of 843 million reads were sequenced with an average of 47 million reads per sample (**Table 1**). An average of 68% of reads was uniquely mapped to the *O. sativa* genome per sample. A principal component analysis also discriminates the transcriptome of non-inoculated plants compared with the inoculated ones as well as the plant responses to each bacterial strain (**Figures 3A, B**). Differential expression analysis yields a total of 4,951 and 5,275 significantly (*p* < 0.01) differentially expressed genes (DEGs) in response to *Bv* and *Pk*, respectively. Comparing leaf and root transcriptomes reveals that there are five and eight times more DEGs detected in leaves in response to *Bv* and *Pk*, respectively, than in roots (**Figures 3C, D**). When comparing the response to *Pk* and *Bv*, a large proportion of DEGs are commonly regulated in leaves (around 50% in response to both strains) contrarily to roots in which the transcriptional regulation appears to be more specific to each inoculated strain. Indeed, only 33% of the DEGs in response to *Bv* are also differentially expressed in response to *Pk*. The proportions of commonly up-regulated DEGs in roots represent 20% and 27% of DEGs in response to *Bv* and *Pk*, respectively.

**Table 1:** Summary of RNAseq data generated for rice transcriptomes.

Sample	Organ	Condition	Total Number of Reads	Number of Uniquely Mapped Reads	Proportion of Mapped Reads (%)
LC1	Leaves	Control	38,533,867	26,161,511	68
LC2	Leaves	Control	44,792,837	29,952,254	67
LC3	Leaves	Control	33,331,123	21,989,917	66
LK1	Leaves	*Paraburkholderia kururiensis*	46,895,520	32,677,603	70
LK2	Leaves	*P. kururiensis*	65,261,206	42,077,779	64
LK3	Leaves	*P. kururiensis*	46,707,369	33,553,685	72
LV1	Leaves	*Burkholderia vietnamiensis*	43,862,789	31,855,527	73
LV2	Leaves	*B. vietnamiensis*	41,034,781	27,022,594	66
LV3	Leaves	*B. vietnamiensis*	49,608,083	35,455,520	71
RC1	Roots	Control	38,522,573	27,000,000	70
RC2	Roots	Control	50,820,144	35,400,000	70
RC3	Roots	Control	44,458,436	30,800,000	69
RK1	Roots	*P. kururiensis*	49,275,052	33,600,000	68
RK2	Roots	*P. kururiensis*	52,634,407	34,700,000	66
RK3	Roots	*P. kururiensis*	49,754,162	30,900,000	62
RV1	Roots	*B. vietnamiensis*	51,314,965	34,700,000	68
RV2	Roots	*B. vietnamiensis*	39,023,620	25,800,000	66
RV3	Roots	*B. vietnamiensis*	57,406,096	39,300,000	68

In order to identify the biological processes transcriptionally regulated by the colonization of both bacterial strains, a GO enrichment analysis was carried out on the commonly regulated DEGs (intersections in **Figures 3C, D**). **Supplementary Figure 3** provides a visualization of the enriched GO terms from the commonly up-regulated or down-regulated DEGs in leaves and roots (complete list of enriched GO terms available in **Supplementary Table 4**). Three main biological processes are commonly regulated during the interaction with both strains in leaves and roots: response to stimuli, and metabolic and also developmental processes. First, stress-related genes are enriched in the commonly DEGs. Indeed, in leaves, the “response to abiotic stimulus” as well as the “response to oxidative stress” terms are enriched in the up-regulated DEGs, whereas defense-related GO terms are enriched in the down-regulated DEGs in both leaves and roots. Also, several hormone-related GO terms are enriched in the DEGs in both leaves and roots. In leaves, GO terms related to auxins and abscisic acid (ABA) response are enriched in the up-regulated genes, while in roots, the up-regulated DEGs are enriched in cytokinines (CK), brassinosteroids (BR), and ethylene response terms. Finally, in roots, down-regulated DEGs are enriched in gibberellic acid (GA) and salicylic acid (SA) response-related terms. The analysis also revealed that metabolic processes are transcriptionally regulated in response to the interaction with both strains: In leaves, the up-regulated DEGs are enriched in the “photosynthesis” and “translation” terms, while the down-regulated DEGs are enriched in “starch metabolic process” and “membrane lipid catabolic process” terms. Furthermore, in leaves, the “iron ion homeostasis” term is enriched in up-regulated DEGs, while the “metal ion transport” term is enriched in the down-regulated DEGs of roots. Although the inoculation of both strains did not significantly impact the biomass of rice plants (**Supplementary Figure 2**), several development-related GO terms are enriched in the DEGs in both leaves and roots. First, in leaves, among others, the “anatomical structure development” term is enriched in the up-regulated DEGs, and the “glucan biosynthetic process” term is enriched in the down-regulated DEGs, which correspond to genes implicated in cellulose and callose synthesis. Finally, root transcriptional response is also enriched in development-related GO terms such as the “xylem development” term enriched in the up-regulated DEGs and the “lignin biosynthetic process” term enriched in the down-regulated DEGs.

Additionally, in order to identify the biological processes specifically induced during the interaction with each strain, we performed a GO term enrichment analysis on the DEGs specifically regulated by each strain (**Supplementary Figures 4** and **5**; **Supplementary Tables 5** and **6**). This analysis revealed that leaves of plants inoculated with *Pk* up-regulate genes related to biosynthetic process and translation, while the interaction with *Bv* induces the up-regulation of genes coding for components of the photosystem II and also the down-regulation of protein folding genes (**Supplementary Figure 4**, genes listed in **Supplementary Table 7**). Interestingly, the up-regulated DEGs in response to each strain are enriched in one hormonal signaling pathway. Indeed, the interaction with *Pk* induced the up-regulation of JA-related genes while cytokinin-related genes are enriched in the up-regulated DEGs in the leaves of *Bv*-inoculated plants. The analysis of the specific root transcriptome also revealed processes specifically induced by each strain. Particularly, the response to *Pk* in roots encompasses the down-regulation of oxidative stress response-related genes and also chitin catabolic process, while the interaction with *Bv* induced the down-regulation of genes involved in defense, JA signaling, and response to stimuli (**Supplementary Figure 5**, corresponding genes in **Supplementary Table 8**).

**Figure 4:**
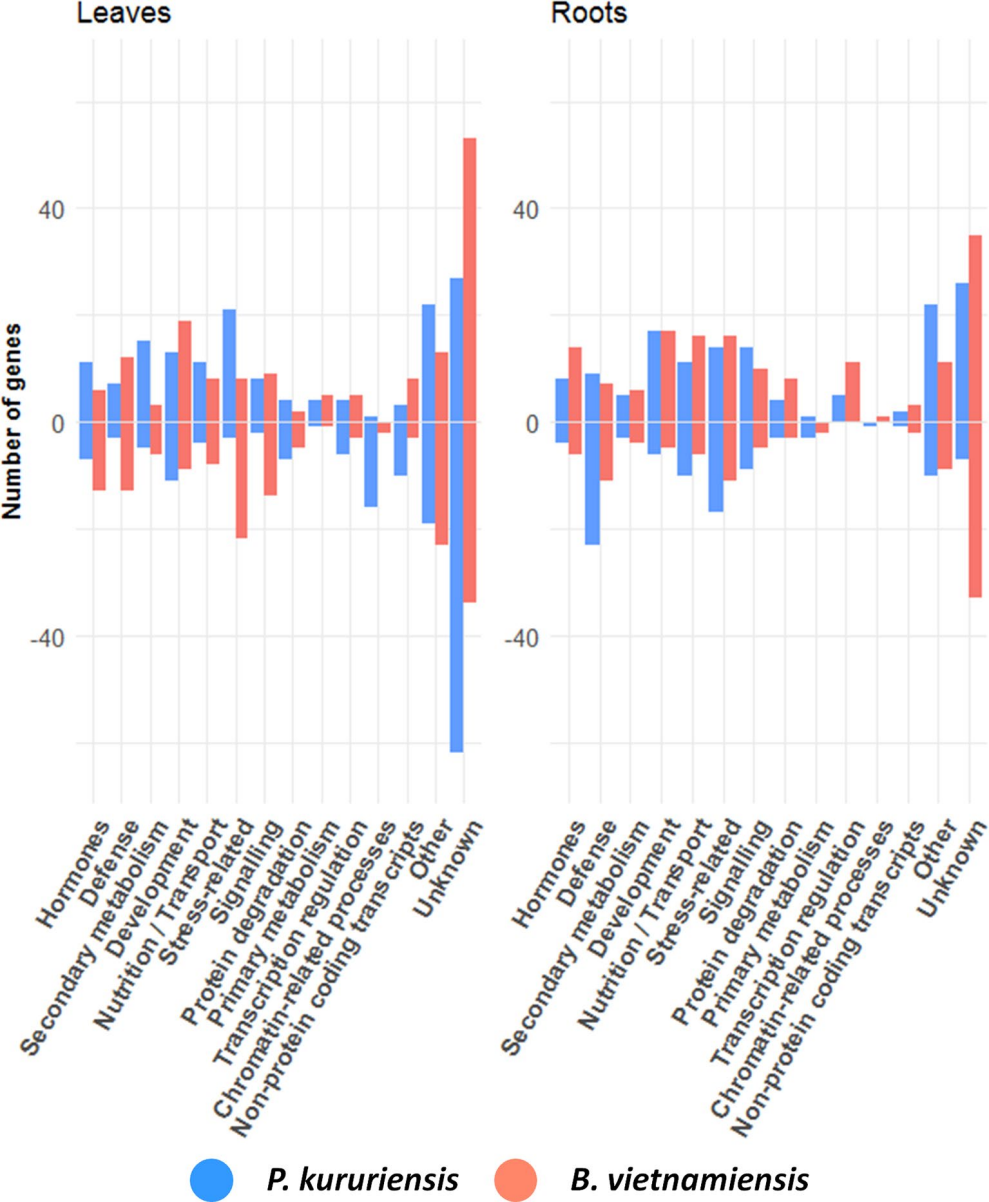
Functional categories of rice DEGs upon *Bv* and *Pk* colonization. Number of DEGs related to functional categories among the top 200 up-regulated and down-regulated DEGs in response to each strain. For each graph, positive or negative numbers correspond to the number of up-regulated or down-regulated genes, respectively. DEGs, differentially expressed genes; *Bv*, *Burkholderia vietnamiensis* TVV75^T^; *Pk*, *Paraburkholderia kururiensis* M130.

In order to deepen the transcriptome analysis, a functional categorization of the top 200 up-regulated and down-regulated DEGs identified in response to each strain was carried out using all available databases for rice annotation (UniProt, Kyoto Encyclopedia of Genes and Genomes (KEGG), RAP-DB, and Oryzabase). The proportion of genes related to each category is presented in **Figure 4** and exemplifies rice-specific responses to the two bacterial strains. In leaves, more stress-related genes as well as genes involved in secondary metabolism are up-regulated in response to *Pk* compared with the response to *Bv*. Also, in response to *Pk*, 16 genes related to chromatin remodeling are down-regulated compared with two in response to *Bv*. Inversely, the inoculation of *Bv* induced the down-regulation in leaves of 22 stress-related genes, whereas only three are down-regulated in response to *Pk*. In roots, twice as much transcription regulation and protein degradation-related genes are up-regulated in response to *Bv* than in response to *Pk*. Conversely, approximately twice as much “nutrition/transport” and signaling-related genes are down-regulated in response to *Pk* than in response to *Bv*.

In order to identify plant key genes that could be related to the observed patterns of root colonization, we focused our analysis on defense (leaves in **Table 2**, roots in **Table 3**) and hormone-related genes (leaves in **Table 4**, roots in **Table 5**). Indeed, these processes are enriched among the strain-specific DEGs (**Supplementary Figures 4** and **5**) and could pinpoint differences in the physiological response of rice following the perception of each strain.

**Table 2:** Defense-related differentially expressed genes (DEGs) in leaves in response to *Paraburkholderia kururiensis* and *Burkholderia vietnamiensis*. Presented genes are part of the top 200 up-regulated and down-regulated significantly DEGs [false discovery rate (FDR) < 0.01]; all results for leaf transcriptome can be found in **Supplementary Table 6**.

Gene ID	Log_2_ fold change		Gene Symbol/Trait Ontology	RAP Annotation
	*P. kururiensis*	*B. vietnamiensis*		
Os05g0537100	1.70	2.44	*WRKY7*	WRKY7
Os07g0539900	−2.17	−1.59	*PR2*	Similar to beta-1,3-glucanase-like protein
Os02g0118875	−1.89	−1.80	NB-ARC	NB-ARC domain-containing protein
Os01g0940800	−1.74	−1.52	*Gns6*	Similar to beta-1,3-glucanase precursor
Os10g0124400	−1.54	−1.67	NB-ARC	Hypothetical conserved gene
Os05g0492600	−1.84	−1.59	*YR48*	Similar to NBS-LRR type resistance protein
Os01g0731100	2.69		*PR*	Similar to pathogen-related protein
Os02g0759400	2.57		*RING1*/Disease resistance	Zinc finger, RING/FYVE/PHD-type domain-containing protein
Os02g0787300	1.88		*MAPKK4*	Mitogen-activated protein kinase kinase 4, defense response
Os02g0626100	1.70		*PAL1*	Similar to phenylalanine ammonia-lyase
Os02g0181300	1.66		*WRKY71*	WRKY transcription factor, defense response
Os12g0520200	1.56		*PAL3*	Similar to phenylalanine ammonia-lyase
Os01g0855600	1.55		*HS1*	Similar to Hs1pro-1 protein
Os11g0227200	−1.57		LRR/Disease resistance	Similar to NBS-LRR disease resistance protein homologue
Os02g0570400	−1.61		*KSL7*/Disease resistance	Similar to Ent-kaurene synthase 1A
Os09g0474000	−1.67		*TFX1*/Disease resistance	bZIP transcription factor, bZIP-1 domain-containing protein
Os01g0859500	−1.73		*LG2*	Similar to basic leucine zipper protein (Ligueless2)
Os05g0375400		2.21	*PR2*, *OsEGL1*	Beta-glucanase precursor
Os03g0320600		1.98	*VQ11*	VQ domain-containing protein
Os08g0170200		1.88		Disease resistance protein domain-containing protein
Os04g0462500		1.75	*GF14b, GF14b, 14-3-3b*	Similar to 14-3-3-like protein GF14-6
Os04g0680800		1.64	*MLO4, MLO4*	Mlo-related protein family protein
Os11g0505300		1.63	*STV11, SOT1*	Sulfotransferase, resistance to rice stripe virus
Os12g0468300		1.63	NB-ARC	Similar to NB-ARC domain-containing protein, expressed
Os02g0251900		1.61	*VQ7*	Similar to tobacco rattle virus-induced protein variant 2
Os04g0118800		1.54	*NB-ARC*	NB-ARC domain-containing protein
Os01g0837000		1.54	*NPR4*, *NPR4*	Ankyrin repeat containing protein
Os11g0604900		1.51	NB-ARC	Similar to NB-ARC domain-containing protein
Os01g0289600		−1.46	*WRKY9*	Similar to WRKY transcription factor 9
Os01g0382000		−1.47	*PR1b*	Similar to pathogenesis-related protein PRB1-2 precursor
Os08g0235800		−1.49	*WRKY25*	Similar to WRKY transcription factor 25
Os10g0569400		−1.50	*Rir1a*	RIR1a protein precursor
Os08g0386200		−1.55	*WRKY69*	WRKY transcription factor 69
Os05g0427400		−1.64	*PAL4*	Similar to phenylalanine ammonia-lyase
Os08g0173600		−1.86	*PROPEP4*/Anti herbivore response	Conserved hypothetical protein
Os07g0117900		−1.90	*NB-ARC*	NB-ARC domain-containing protein
Os06g0244000		−2.09	*AAMT*	Similar to anthranilic acid methyltransferase 3
Os04g0205200		−2.09	NB-ARC	NB-ARC domain-containing protein
Os03g0195100		−2.16	*ALD1*	Putative aminotransferase, response against blast fungus
Os12g0154800		−2.31	*GLP12-2*	Germin-like protein 12-2, disease resistance
Os12g0154700		−2.91	*GLP12-1*	Germin-like protein 12-1, disease resistance

**Table 3:** Defense-related differentially expressed genes (DEGs) in roots in response to *Paraburkholderia kururiensis* and *Burkholderia vietnamiensis*. Presented genes are part of the top 200 up-regulated and down-regulated significantly DEGs [false discovery rate (FDR) < 0.01]; all results for root transcriptome can be found in **Supplementary Table 7**.

Gene ID	Log_2_ fold change		Gene Symbol/Trait Ontology	RAP Annotation
	*P. kururiensis*	*B. vietnamiensis*		
Roots				
Os08g0332600	1.07	0.74	NB-ARC	Disease resistance protein domain-containing protein
Os08g0189500	−1.15	−1.57	*OsGLP8-6*	Germin-like protein 8-6, disease resistance
Os08g0231400	−1.15	−1.62	*GLP8-12*	Germin-like protein 8-12, Disease resistance
Os10g0537800	−1.76	−1.80	*AP77*	Peptidase aspartic, catalytic domain-containing protein
Os12g0500500	−1.72	−1.84	NB-ARC	NB-ARC domain-containing protein
Os01g0508500	−2.07	−1.89	*RH2*/*NRR repressor homolog 2*	Conserved hypothetical protein
Os02g0807900	−1.12	−2.17	*WAK21*	Conserved hypothetical protein
Os09g0417600	−1.92	−2.64	*WRKY76*	WRKY transcription factor, transcriptional repressor
Os03g0195100	−1.91	−2.65	*ALD1*	Putative aminotransferase, response against blast fungus
Os09g0417800	−2.49	−2.66	*WRKY62*	WRKY transcription factor, transcriptional repressor
Os01g0508100	−2.07	−2.83	*RH3*/*NRR repressor homolog 3*	Ferritin/ribonucleotide reductase-like family protein
Os06g0105100	−2.75	−3.16	*WRKY86*	Similar to legumain
Os05g0247800	−3.47	−3.74	*XIP*	Glycoside hydrolase, family 18 protein
Os08g0332600	1.07		NB-ARC	Disease resistance protein domain-containing protein
Os08g0446200	0.75		*OsPEPR1*	Similar to receptor-like protein kinase precursor
Os12g0448900	0.74		*PIOX, RalphaO*	Fatty acid alpha-dioxygenase family
Os09g0127300	0.71		*CCR17*	NAD(P)-binding domain-containing protein
Os12g0199100	0.70		NB-ARC	NB-ARC domain-containing protein
Os07g0273700	0.69		*WRKY123*	Disease resistance protein domain-containing protein
Os01g0269800	0.68		*NB-ARC*	NB-ARC domain-containing protein
Os03g0411100	0.64		*OsHAP2E*	Heme activator protein, biotic and abiotic resistances
Os11g0152700	0.43		*OsbZIP79*	Similar to transcription factor HBP-1b(C38) (fragment)
Os02g0626400	0.41		*PAL8*	Phenylalanine ammonia-lyase (EC 4.3.1.5)
Os01g0713200	−1.14		*Gns10*	Similar to beta-glucanase
Os02g0629800	−1.15		*OsPR12/DEFL7*	Similar to defensin precursor
Os05g0554000	−1.19		*MATE2*	Similar to cDNA clone:001-123-D07, full insert sequence
Os12g0555200	−1.20		*PBZ1*	Similar to Probenazole-inducible protein PBZ1
Os05g0247100	−1.24		*HI-XIP*/Insect resistance	Similar to glycosyl hydrolases family 18
Os07g0129200	−1.26		*PR1a*	Pathogenesis-related 1a protein
Os07g0127500	−1.31		*PR1-72*	Similar to PR-1a pathogenesis related protein (Hv-1a) precursor
Os02g0570700	−1.37		*Cyp71Z7*	Cytochrome P450 family protein
Os01g0947000	−1.42		Disease resistance	Similar to beta-1,3-glucanase precursor
Os02g0605900	−1.45		*CHIT6*	Similar to chitinase (EC 3.2.1.14) A
Os12g0437800	−1.62		*Sci1, PR6*	Similar to MPI
Os11g0700900	−1.67		C10923/Chitinase	Glycoside hydrolase
Os01g0940700	−1.85		*PR2, Glu1*	Similar to glucan endo-1,3-beta-glucosidase
Os07g0127700	−1.98		Disease resistance	Similar to pathogenesis-related protein class 1
Os12g0628600	−1.99		*PR5*	Similar to thaumatin-like pathogenesis-related protein 3 precursor
Os07g0663700	−2.19		*SDR110C-MI3*/Disease resistance	Similar to oxidoreductase
Os08g0518900	−2.27		*C10122*/Disease resistance	Chitinase (EC 3.2.1.14)
Os03g0130300	−2.54		DEF8	Similar to Cp-thionin
Os11g0701600	−2.84		*Chitinase*	Glycoside hydrolase, catalytic domain-containing protein
Os04g0316200	−2.92		Gnk2-domain protein	Protein of unknown function DUF26 domain-containing protein
Os11g0701800	−3.16		*OsRIXI*/Disease resistance	Chitinase (EC 3.2.1.14) III
Os07g0129300	−3.67		*PR1a*	Similar to PR1a protein
Os11g0701000	−4.67		*Chib3H-c*	Class III chitinase homologue (OsChib3H-c)
Os04g0289500		3.58	PR1	Allergen V5/Tpx-1-related domain-containing protein
Os12g0491800		2.15	*KSL10*	Similar to Ent-kaurene synthase 1A
Os08g0374000		0.99	*OsBetvI*	Bet v I allergen family protein
Os10g0163040		0.96	NB-ARC	Similar to Blast resistance protein
Os08g0261000		0.91	NB-ARC	NB-ARC domain-containing protein
Os09g0356000		0.91	*SIRK1*	Similar to OsD305
Os01g0859500		0.79	*LG2*	Similar to Basic leucine zipper protein (Liguleless2)
Os09g0517200		−1.65	NB-ARC	Hypothetical conserved gene
Os05g0478700		−1.67	*WRKY84*	Hypothetical conserved gene
Os03g0335200		−1.68	*WRKY79*	Similar to WRKY1 (WRKY transcription factor 17)
Os11g0117500		−1.75	*WRKY40*	DNA-binding WRKY domain-containing protein
Os06g0649000		−1.81	*WRKY28*	PAMP-responsive transrepressor
Os04g0635500		−1.93	Blast/SA induced	Similar to Wound induced protein (fragment)
Os11g0154500		−2.09	*Nac17*/Disease Resistance	Blast disease-responsive transcription factor, disease resistance
Os11g0462500		−2.66	NB-ARC	Similar to NB-ARC domain-containing protein
Os05g0248200		−4.65	*Chitinase*	Glycoside hydrolase, family 18 protein
Os04g0375300		−6.34	*rNBS56*	Similar to NBS-LRR protein (fragment)
Os06g0279900		−7.11	NB-ARC	Similar to NB-ARC domain-containing protein, expressed

**Table 4:** Hormone-related differentially expressed genes (DEGs) in leaves in response to *Paraburkholderia kururiensis* and *Burkholderia vietnamiensis*. Presented genes are part of the top 200 up-regulated and down-regulated significantly DEGs (false discovery rate (FDR) < 0.01); all results for leaf transcriptome can be found in **Supplementary Table 6**.

Gene ID	Log_2_ fold change		Gene Symbol/Function	RAP Annotation
	*P. kururiensis*	*B. vietnamiensis*		
Gibberellic acid				
Jasmonate				
Auxin				
Ethylene				
Abscisic acid				
Cytokinine				
Brassinosteroid				
Os06g0729400	1.85	2.74	*GASR8*	Similar to gibberellin-regulated protein 2 precursor
Os09g0470500	−1.49		*Hox4*	Homeodomain leucine zipper protein
Os03g0352450	−1.60		*INO80*, *CHR732*/GA signaling	Hypothetical conserved gene
Os08g0560000	−1.66		*GA20ox7*	Similar to gibberellin 20 oxidase 2
Os01g0757200		2.11	*GA2ox3*	GA 2-oxidase3, GA metabolism
Os06g0110000		1.59	*KAO*	Similar to DWARF3 (fragment)
Os02g0570400		−1.61	*KS7*	Similar to Ent-kaurene synthase 1A
Os04g0301500	1.88	−1.65	*RERJ1*	Helix-loop-helix DNA-binding domain-containing protein
Os03g0767000	2.11		*AOS1*	Allene oxide synthase (CYP74A1), Biosynthesis of jasmonic acid (JA)
Os01g0705700	1.84		*MYL1*	Similar to transcription factor ICE1 (Inducer of CBF expression 1)
Os10g0392400	1.49		*JAZ12*	Tify domain-containing protein
Os05g0439100		1.46	*MYC7E*	Similar to transcription factor MYC7E (fragment)
Os03g0181100		−1.97	*JAZ10*	Tify domain-containing protein
Os01g0643300	2.44	2.29	*PIN3A*	Auxin efflux carrier protein, auxin transport
Os12g0601300	2.81		*IAA30*	Similar to auxin-responsive protein (Aux/IAA) (fragment)
Os07g0182400		1.93	*IAA24*	AUX/IAA protein family protein
Os02g0523800		1.72	*IPK2*	Inositol polyphosphate kinase, auxin signaling
Os06g0323100		−1.53	IAA Methylase	Similar to H1005F08.18 protein
Os06g0671600		−1.69	*Small auxin-up RNA 26*	Beta tubulin, autoregulation binding site domain-containing protein
Os05g0528600		−2.02	*YUCCA2*	Flavin monooxygenase-like enzyme, auxin biosynthesis
Os09g0522000	2.94	−1.91	*DREB1B*	Similar to dehydration-responsive element-binding protein 1B
Os09g0451000	2.19	1.64	*ACO2*	ACC oxidase, ethylene biosynthesis
Os04g0572400	4.19		*DREB1E*	Similar to CRT/DRE binding factor 1
Os02g0677300	3.41		*DREB1G*	Similar to CRT/DRE binding factor 1
Os10g0562900	2.87		*DERF12*	Non-protein coding transcript
Os08g0474000	2.64		*DERF3*	Similar to AP2 domain-containing protein RAP2.6 (fragment)
Os02g0654700	2.27		*BIERF3*	AP2/ERF family protein, abiotic stress response
Os09g0522200	1.83		*DREB1A*	DRE-binding protein 1A
Os07g0155600	−1.53		*EIN2*	Nramp ion-transporter family protein, ethylene signaling pathway
Os07g0410300		4.78	*ERF136*	Conserved hypothetical protein
Os07g0410700		1.93	*DERF2*	Similar to Ethylene-responsive element binding protein 1
Os01g0797600		1.66	*BIERF2*	AP2/ERF family protein, stress signaling
Os02g0676800		−1.94	*ERF20*	Similar to dehydration responsive element binding protein 1E (DREB1E protein)
Os06g0493100		−2.31	*bphi008a*	Conserved hypothetical protein
Os02g0636600	−2.27	−1.81	*GEM*/ABA-related	GRAM domain-containing protein
Os02g0703600	3.07		*ABA8OX1*	Similar to abscisic acid 8′-hydroxylase 1
Os07g0569100	1.86		*REM4.1*	Remorin protein, coordination of interlink between ABA and BR signaling
Os07g0281800		−1.57	ABA synthesis	Similar to aldehyde oxidase-2
Os02g0182100	−1.80		RR24	B-type response regulator, cytokinin signaling
Os11g0143300		−1.71	RR9	A-type response regulator, cytokinin signaling
Os01g0952500		−1.77	RR4	A-type response regulator, cytokinin signaling
Os08g0460600		−1.89	CKX11	Similar to cytokinin dehydrogenase 11
Os01g0197100	1.72		*dwf2*, *SMG11*	Cytochrome P450, brassinosteroids biosynthesis, Regulation of plant architecture
Os09g0409950	−1.48		*BRI1-interacting protein 120*	Hypothetical conserved gene
**Strigolactone**				
Os01g0935400	1.62		*AtLBO* ortholog	2OG-Fe(II) oxygenase domain-containing protein
**Salicylic acid**				
Os04g0581100		−2.01	*S3H*/SA conjugation	2OG-Fe(II) oxygenase domain-containing protein

**Table 5:** Hormone-related differentially expressed genes (DEGs) in roots in response to *Paraburkholderia kururiensis* and *Burkholderia vietnamiensis*. Presented genes are part of the top 200 up-regulated and down-regulated significantly DEGs [false discovery rate (FDR) < 0.01]; all results for root transcriptome can be found in **Supplementary Table 7**.

Gene ID	Log_2_ fold change		Gene Symbol/Function	RAP Annotation
	*P. kururiensis*	*B. vietnamiensis*		
Jasmonate				
Auxin				
Cytokinine				
Ethylene				
Brassinosteroids				
Gibberellic acid				
Abscisic acid				
Salicylic acid				
Os01g0370000	0.87	0.96	*OPR9*	NADH:flavin oxidoreductase/NADH oxidase
Os05g0362100	0.57		JA synthesis	Similar to protein MFP-b
Os08g0509100	−2.07		*LOX8*	Similar to lipoxygenase, chloroplast precursor (EC 1.13.11.12)
Os03g0181100		−1.62	*JAZ10*	Tify domain-containing protein
Os03g0741100		−1.93	*bHLH148*/JA responsive	Basic helix-loop-helix transcription factor
Os03g0180800		−2.01	*JAZ9*	TIFY domain-containing transcriptional regulator
Os02g0228900	0.58		*IAA7*	Similar to auxin-responsive protein IAA18
Os11g0523800	0.49		*ARF1*	Similar to isoform 3 of auxin response factor 23
Os02g0305950		−2.92	*SAUR PROTEIN 7*	Similar to calmodulin binding protein
Os08g0529000		−7.15	*PIN5b*	Auxin efflux carrier, auxin homeostasis
Os04g0673300	0.66	0.94	*RR6*	A-type response regulator, cytokinin signaling
Os04g0556500	−1.35	−1.64	*cZOGT1*	*cis*-zeatin-*O*-glucosyltransferase
Os07g0449700	1.55		*RR7*	A-type response regulator, cytokinin signaling
Os10g0483500	0.66		*CKX3*	FAD linked oxidase, N-terminal domain-containing protein
Os08g0460600		1.29	*CKX11*	Similar to cytokinin dehydrogenase 11
Os11g0143300		1.02	*RR9*	A-type response regulator, cytokinin signaling
Os12g0139400		1.00	*RR10*	A-type response regulator, cytokinin signaling
Os02g0830200		0.81	*RR3*	A-type response regulator, cytokinin signaling
Os05g0591600		−1.76	*LOGL8*/CK synthesis	Similar to carboxy-lyase
Os06g0573900	1.31	2.12	*ACO*	Similar to 1-aminocyclopropane-1-carboxylic acid oxidase
Os02g0771600	1.03	1.06	*ACO3*	Similar to 1-aminocyclopropane-1-carboxylate oxidase
Os04g0610900	0.77	0.93	*CTR3*	Similar to EDR1
Os03g0341000	−1.79	−1.99	*ERF66*	Similar to AP2 domain-containing protein RAP2.2
Os07g0674800	−2.11	−3.02	*ERF67*	Similar to AP2 domain transcription factor EREBP
Os01g0313300	−1.36	−2.79	*ERF68*	Similar to EREBP-3 protein (fragment)
Os07g0575000	1.69		*ERF6*	ERF, DNA-binding domain-containing protein
Os05g0155200	0.49		*ERS2*	Similar to ethylene receptor
Os05g0361700	−1.07		*ERF61*	Similar to EREBP-2 protein (fragment)
Os06g0586000	−1.31		ETH responsive	Conserved hypothetical protein
Os03g0183200		1.25	*ERF69*	Similar to AP2 domain-containing protein, expressed
Os05g0316800		1.03	*ERF56*	Similar to ethylene-responsive transcription factor 9
Os10g0390800		0.75	*EBL1*	Similar to ethylene-responsive transcription factor 3 (EREBP-3)
Os09g0522000		−2.71	*DREB1B*	Similar to dehydration-responsive element-binding protein 1B
Os02g0677300		−3.39	*DREB1G*	Similar to CRT/DRE binding factor 1
Os11g0289700	1.28		BR synthesis	Cytochrome P450 family protein
Os04g0641700	−1.32		*ILI1*	Similar to H0423H10.4 protein
Os09g0441400		−1.02	BR synthesis	Similar to elicitor-inducible cytochrome P450
Os05g0560900	−1.15	−1.73	*GA2ox8*	Similar to gibberellin 2-beta-dioxygenase
Os06g0729400	0.55		*GASR8*	Similar to Gibberellin-regulated protein 2 precursor
Os05g0158600		−1.44	*GA2ox1*	Similar to OsGA2ox1
Os03g0437200	−1.99	−1.67	*Bsr-d1*	Abscisic acid-induced antioxidant defense
Os02g0255500	0.42	0.72	*PYL3*	Similar to extensin (fragment)
Os04g0581100	−2.06		*S3H*/SA conjugation	2OG-Fe(II) oxygenase domain-containing protein
Os11g0259700		2.02	SA Conjugation	Similar to SAM-dependent carboxyl methyltransferase
**Strigolactones**				
Os03g0203200		0.69	*dwf14*	Strigolactone receptor, strigolactone perception, regulation of shoot branching

In roots, 13 defense-related genes are commonly regulated, while 33 and 18 are specifically *Pk* and *Bv* regulated (**Table 3**). Additionally, 12 hormone-related genes (implicated in JA, CK, ethylene, GA, and ABA syntheses or signaling) are commonly regulated in response to both strains, while 14 and 19 hormone-related genes are *Pk* and *Bv* regulated, respectively (**Table 5**). The latest encompasses genes implicated in auxins, BR, SA, and strigolactone synthesis, or signaling. In leaves, 41 defense-related genes are regulated, six being commonly regulated, while 11 and 24 are specifically induced by *Pk* and *Bv*, respectively (**Table 2**). Also hormone-related DEGs are detected in leaves; six genes implicated in GA, JA, auxins, ethylene and ABA synthesis, transport, or signaling are commonly regulated. Finally, in response to each strain, 20 hormone-related specific DEGs are detected. Interestingly, only the interaction with *Pk* induced the regulation of BR- and SL-related genes, while only *Pv* induced the regulation of a SA-related gene. Other hormonal pathways, such as CK, ABA, ethylene, JA, auxins, and GA, are modulated by each strain but induced the regulation of different genes (**Table 4**).

### Validation of RNAseq Data by qCPR

**Figure 5:**
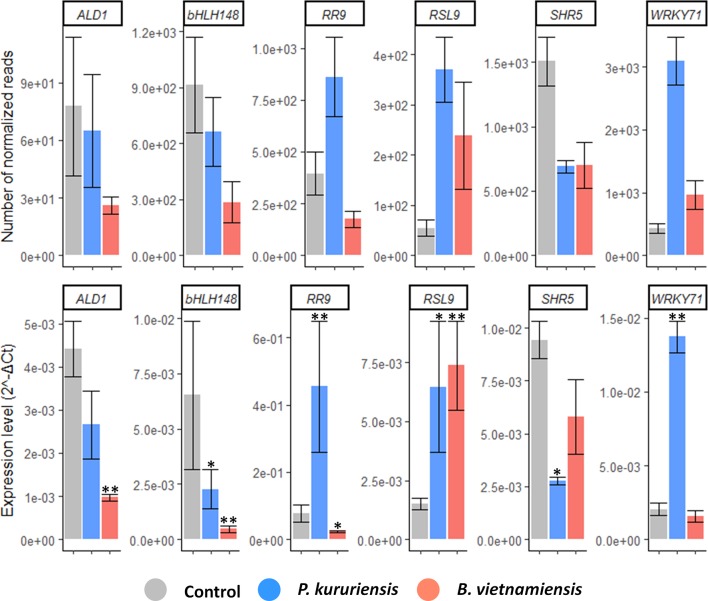
Confirmation of genes regulated upon *Bv* and *Pk* colonization. Gene expression quantified by RNAseq (top) and quantitative reverse transcriptase–polymerase chain reaction (qRT-PCR; bottom). For RNAseq, the data presented are the mean count of normalized reads. For qRT-PCR, transcript levels were normalized to that of the reference gene EF 1α (Os03g0177400). Stars represent significant differences of mean expression compared with the control condition according to *post-hoc* test on a generalized linear model. * corresponds to *p* < 0.05 and ** to *p* < 0.0001. Error bars represent the standard deviation (*n* = 3). *Bv*, *Burkholderia vietnamiensis* TVV75^T^; *Pk*, *Paraburkholderia kururiensis* M130; DEGs, differentially expressed genes.

To validate the RNAseq data, we selected genes implicated in defense, hormone signaling, or development regulated at 7 dpi by one or both strains (**Supplementary Table 9**). We measured the level of expression of the selected genes by qRT-PCR in an independently conducted experiment. According to RNAseq analysis, in leaves, two defense-related genes are specifically regulated by each strain: *ALD1* is specifically down-regulated in response to *Bv*, and *WRKY71* is specifically up-regulated in response to *Pk*. Also, two hormone-related genes were detected as specifically regulated by each strain; namely, *bHLH148*, a JA signaling component, is down-regulated in roots inoculated with *Bv*; while *RR9*, a CK signaling component, is up-regulated in the leaves of *Pk*-inoculated plants. Finally, in roots, *RSL9* is up-regulated and *SHR5* is down-regulated in response to both strains. Gene expression changes obtained through qPCR analysis demonstrated a pattern similar to RNAseq (**Figure 5**) except for the over-expression of *RR9* in response to *Pk*, which was not detected as significant by DESeq2.

### Temporal Analysis of Strain-Specific Marker Genes

Following the confirmation of strain-specific transcriptional regulations, we wanted to identify genes that are differentially expressed in both conditions but with opposite regulations. Among all DEGs detected in roots and leaves, only three DEGs are potential differential markers: *RERJ1* (Os04g0301500), a JA-responsive gene; *ATL15* (Os01g0597600), a putative amino acid transporter; and *DREB1B* (Os09g0522000), a drought-responsive gene. Noteworthy, these three genes are only detected as DEGs in leaves and follow the same pattern of expression, as they are up-regulated in response to *Pk* and down-regulated in response to *Bv*.

**Figure 6:**
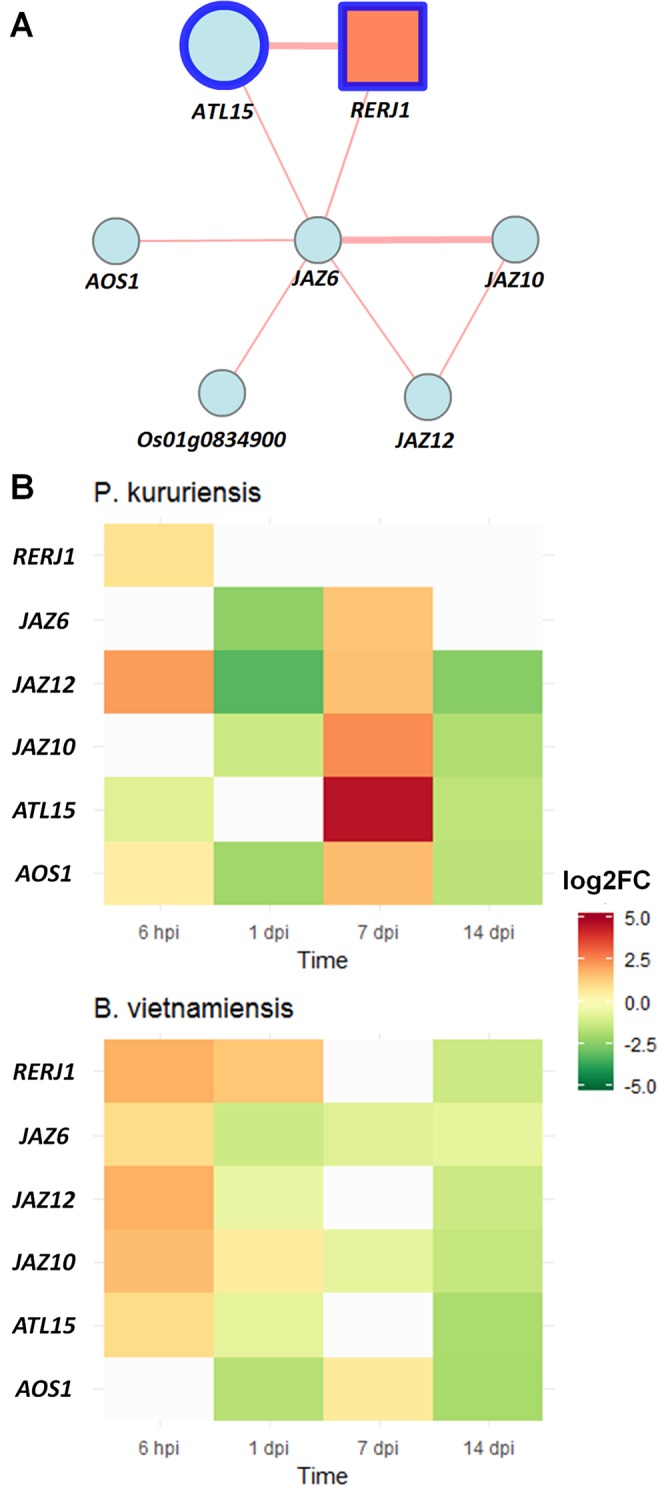
JA-related co-expression network transcriptional regulations induced by *Pk* and *Bv* root colonization. **(A)** Co-expression network of differential markers (circled in blue); edges’ width is proportional to the co-expression ratio between each gene according to RiceFREND database. **(B)** Gene expression dynamics of the co-expression network in response to *Pk* and *Bv* quantified by qRT-PCR. Transcript levels were normalized to that of reference gene EF 1α (Os03g0177400). Values between 0.5 and −0.5 are colored in gray. Data presented are the mean of log2 fold change (*n* = 3). JA, jasmonic acid; *Bv*, *Burkholderia vietnamiensis* TVV75^T^; *Pk*, *Paraburkholderia kururiensis* M130.

Among these three genes, two of them, *ATL15* and *RERJ1*, are part of a co-expression network recovered from RiceFREND database (Sato et al., 2013), which contains four JA-related genes (*JAZ6*, *10*, *12*, and *AOS1*) (**Figure 6A**). As JA is one of the main phytohormones implicated in defense (Pozo et al., 2004), we further wanted to know if the whole co-expression network could act as a differential marker of the response to each bacterial strain. In order to describe the transcriptional regulation of this co-expression network throughout the establishment of the interaction, we produced a transcriptional kinetic of rice leaves tissues at 6 hpi, 1 dpi, 7 dpi, and 14 dpi and analyzed all six genes by qRT-PCR.

First, at 7 dpi, we can confirm that depending on the inoculated strains, the regulation of the co-expression network is opposite (**Figure 6**; **Supplementary Table 10**). On the one hand, *JAZ6*, *JAZ10*, *JAZ12*, *ATL15*, and *AOS1* are up-regulated following the inoculation with *Pk*; and on the other hand, *JAZ6* and *JAZ10* are down-regulated in response to *Bv*. Interestingly, and in sharp contrast to the 7 dpi response, for the short-term response (6 hpi), we observed the up-regulation of five out of the six genes of the co-expression network in rice leaves after inoculation with *Bv*, whereas only *JAZ12* is up-regulated in response to *Pk*. Then, the whole network appears to decline for the rest of the kinetics in response to *Bv*. Eventually, at 14 dpi, the expression levels of the six genes are quite comparable between the two conditions being all down-regulated compared with non-inoculated controls.

## Discussion

The aim of this study was to describe the transcriptional regulations induced by rice following the perception of naturally associated beneficial bacteria. Additionally, we took advantage of the particular phylogenetic organization of the *Burkholderia s.l.* genus to compare the responses of plants with two closely related beneficial species with different phylogenetic backgrounds in terms of ecology.

### *P. kururiensis* M130 and *B. vietnamiensis* TVV75 Differentially Colonize Roots of *O. sativa* Cultivar Nipponbare

We first observed that both bacteria were able to efficiently colonize the rice Nipponbare roots. The amount of culturable bacterial cells associated with rice roots appears to be coherent with the literature, as Compant et al. (2010) described that generally a range between 10^7^ and 10^9^ cfu·g^−1^ of root fresh weight is found colonizing roots both externally and internally. The same goes for the endophytic population, which generally ranges between 10^5^ and 10^7^ cfu·g^−1^. The decrease of the bacterial population size between 7 and 14 dpi may be due to the increase of root biomass by the formation of newly emerged roots, which are not importantly colonized at least in the time of our experiments. However, by comparing the colonization of the two strains, two main differences can be observed in the dynamic of root colonization. First, *Pk* forms a significantly bigger population while colonizing rice roots surface than does *Bv* at every time postinoculation. Second, the dynamic of the endophytic population of both strains is very different, as the number of endophytic *Pk* cells declines between 7 and 14 dpi which is not the case for *Bv*. This could be due to the fact that the endophytic colonization by *Pk* is restricted by the plant throughout time in contrast to *Bv*, which maintains its population size. From this observation, two hypotheses can be proposed: Either the plant is not able to control the colonization by *Bv*, or this strain is more efficiently colonizing the newly emerged roots.

Both the maturation zone and the area of lateral root emergence were identified as hotspots for *Pk* and *Bv* colonization. A similar area of colonization was identified for *Paraburkholderia phytofirmans* strain PsJN in grape plants (*Vitis vinifera* L.) (Compant et al., 2005) as well as for *B. vietnamiensis* MGK3, which also intensively colonizes the same areas of the roots of rice plants (Govindarajan et al., 2008). Root exudates, which contain essential nutrients for microbes, are released in the lateral root emergence zones (Badri and Vivanco, 2009). This may aid colonization and allow the possible entry of bacteria *via* mechanisms such as “crack entry” into the internal tissues (Hardoim et al., 2008).

Both strains were observed massively colonizing the maturation zone, on both the outside and even the inside of some root hair cells (**Supplementary Figure 1**). The accumulation of bacterial cells in the root hair zone has already been described for *Pk* (Mattos et al., 2008), and it was proposed to be a common hotspot for rhizobacterial colonization (Compant et al., 2010), as it is correlated to a higher local exudate concentration (Gamalero et al., 2004). Apparent intracellular colonization of epidermal cells was observed for both species but more frequently for *Bv*. In the case of *Bv*, the fact that the vacuole is still intact when observing through the colonized epidermic cell offers evidence that the cell is still living (**Figure 2D**). Also, as the bacterial cells seem to have passed through the cell wall, the fluorescence signal appears to be cytoplasmic. Intracellular colonization of rice root epidermal cells by bacteria was also observed during the interaction with *Azoarcus* BH72 (Reinhold-Hurek et al., 2006) as well as during the colonization of ryegrass roots by *Paraburkholderiabryophila* Ha185 (Hsu et al., 2018). Furthermore, intracellular colonization of root hair cells was also observed by Prieto et al. (2011). They showed by confocal microscopy that *Pseudomonas putida* PICP2 and *Pseudomonas fluorescens* PICF7 are able to colonize root hair cells of olive trees (*Olea euroapea* L.) and subsequently move into epidermal cells. This observation led them to propose a new route of entry in intern plant tissues for endophytic bacteria, which starts with the colonization of single root hairs.

### Root Colonization Induces More Transcriptional Regulations in Aerial Parts Than in Roots

The analysis of root and leaf transcriptomes revealed that colonization of rice roots by both strains induced more transcriptional regulations in leaves than in roots (**Figure 3B**). This could be due to the fact that the inoculated plants were harvested at 7 dpi and therefore at an established state of the interaction between bacteria and rice. In contrast, previous studies revealed that the inoculation of beneficial rhizobacteria induced the regulation of at least 1,000 genes in rice roots at earlier time points after inoculation (Drogue et al., 2014; Brusamarello-Santos et al., 2019; Rekha et al., 2018). Moreover, compared with the only study that analyzed the leaf transcriptional response of rice to the inoculation by beneficial rhizobacteria that retrieved only 2,414 DEGs at early stages of the interaction (Wu et al., 2018), when our study identified data of at least 4,000 DEGs in leaves in response to both strains.

### Roots Trigger Contrasted Transcriptional Response Depending on the Inoculated Strain

In response to the colonization by both strains, transcriptional reprogramming of defense-related genes occurs in rice roots (**Table 3**). Only 13 of them appeared commonly regulated in the same way. First, three *WRKY* (*62*, *76*, and *86*),genes are commonly down-regulated; interestingly, those three genes are negative regulator of defense (Peng et al., 2008; Yokotani et al., 2013)—putatively for *WRKY86* (Choi et al., 2017). Also, two negative regulators of *NH1*, the rice *NPR1* homolog of the major SA response regulator (Yuan et al., 2007; Chern et al., 2012), namely, *RH2* and *RH3*, are also down-regulated (Chern et al., 2014). Taken together, as these genes are described as negative regulators of defense, their down-regulation may reflect a common defense response towards root colonizing bacteria.

The remaining 51 genes were differentially regulated in response to each strain (33 responding to *Pk* and 18 to *Bv*, respectively). Indeed, roots colonized with *Pk* induce the down-regulation of 11 pathogenesis-related (PR) genes as well as five chitinases and two xylanase inhibitors. One of the down-regulated PR genes is *PBZ1* (Os12g0555200), and it is described as a defense marker in leaves, but it is also up-regulated in response to root invasion by *Magnaporthe oryzae* (Marcel et al., 2010). Moreover, *bZIP79*, which is described as a phytoalexin synthesis suppressor (Miyamoto et al., 2015), is up-regulated. On the other hand, roots colonization by *Bv* induce the down-regulation of only one chitinase, whereas two PR genes are up-regulated, of which one gene, *BetvI*, is targeted by parasitic nematode to suppress root defense (Chen et al., 2018). Furthermore, four *WRKY* genes of which two are thought to encode negative regulators (*WRKY28* (Chujo et al., 2013) and *WRKY79* (Choi et al., 2017)) and *WRKY40*, which is up-regulated in striga-resistant rice roots (Swarbrick et al., 2008), are here down-regulated. Taken together, these results show that the colonization of rice roots by *Pk* is associated with a down-regulation of *PR* genes, while the colonization by *Bv* is associated with the down-regulation of defense-suppressing *WRKY* genes. This transcriptional regulation of defense-related genes could be the consequence of the more invasive colonization pattern of *Bv* cells described above.

As previously described (Vacheron et al., 2013; Drogue et al., 2014; Rekha et al., 2018), the inoculation of beneficial rhizobacteria induces important hormone-related transcriptional regulation in rice roots (**Table 5**). Similarly, the inoculation of *Pk* and *Bv* induced the regulation of genes encoding for signaling components of CK and ethylene. Particularly, both bacterial strains induced the up-regulation of two genes encoding for ACO, which are enzymes implicated in the synthesis of ethylene (Ravanbakhsh et al., 2018). Interestingly, in response to the inoculation of other beneficial bacteria, genes coding for ACOs were down-regulated in rice roots (Drogue et al., 2014; Brusamarello-Santos et al., 2019). Also, important transcriptional regulations of ethylene responsive factors (ERFs) occur in response to both strains. In the same way, cytokinine signaling is impacted as both conditions induce the up-regulation of at least two *RR* genes and one cytokinine oxidase encoding gene (Tsai et al., 2012); however, it is not the same genes that are regulated by each bacterium.

### Systemic Responses Associated With Root Inoculation Suggest a Time Shift in Defense Response Between the Two Species

Interestingly, the RNAseq analysis revealed that the inoculation of rhizobacteria induced major transcriptional regulations in leaves (**Tables 2** and **4**; **Supplementary Table 5**). As previously described (Persello-Cartieaux et al., 2003; Verhagen et al., 2004; Campos-Soriano et al., 2012), the colonization of beneficial microbes induces transcriptional regulations of defense-related genes in the leaves of the inoculated plants. Both strains induced the up-regulation of *WRKY7* as well as the down-regulation of three putative R genes and two PR genes. *Pk* induced the up-regulation of *WRKY71*, which is known to confer enhanced disease resistance to *Xoo* (Liu et al., 2007) and the down-regulation of *TFX1*, which is known as a susceptibility gene for *Xoo* (Sugio et al., 2007). On the other hand, *Bv* induces the up-regulation of five R genes: three (*NB-ARC*, *MLO4*, and *STV11*) (Wang et al., 2014), *NPR4*, as well as the down-regulation of three *WRKY* genes (*9*, *25*, and *69*), of which two are SA responsive (Liu et al., 2007; Choi et al., 2017), and also the down-regulation of *ALD1*, a basal immunity regulator needed for the accumulation of SA (Jung et al., 2016).

It is also in this organ only that differential markers have been detected, some of which designated the JA signaling pathway as a putative marker of the interaction with the two bacterial species. Also, interestingly, in contrast with the regulation of JA-related genes, several GA-related genes seem to be down-regulated in response to *Pk* and up-regulated in response to *Bv*. This antagonism between JA and GA has been described (Yang et al., 2012; Yimer et al., 2018) and proposed as one of the ways for plants to fine-tune the balance between growth and defense (Huot et al., 2014). Nonetheless, this apparent contrast in terms of JA-related genes transcriptional regulation at 7 dpi may only be a consequence of a delayed JA systemic signaling in response to *Pk* compared with the response to *Bv*, which induced the up-regulation of the co-expression network as soon as 6 hpi. As JA-induced plant defenses have been proposed to contribute to the restriction of endophytic colonization in grasses (Miché et al., 2006), this temporal shift in the induction of JA-related genes between the responses to the two bacterial strains could be associated with a delayed JA-induced defense signal. We propose the following interpretation: the perception of *Bv* induces very early (6 hpi) the up-regulation of JA-related genes in leaves to restrict the colonization, whereas in response to *Pk*, this JA signal happens at 7 dpi and results in the decrease of the bacterial population (**Figure 1A**).

**Figure 7:**
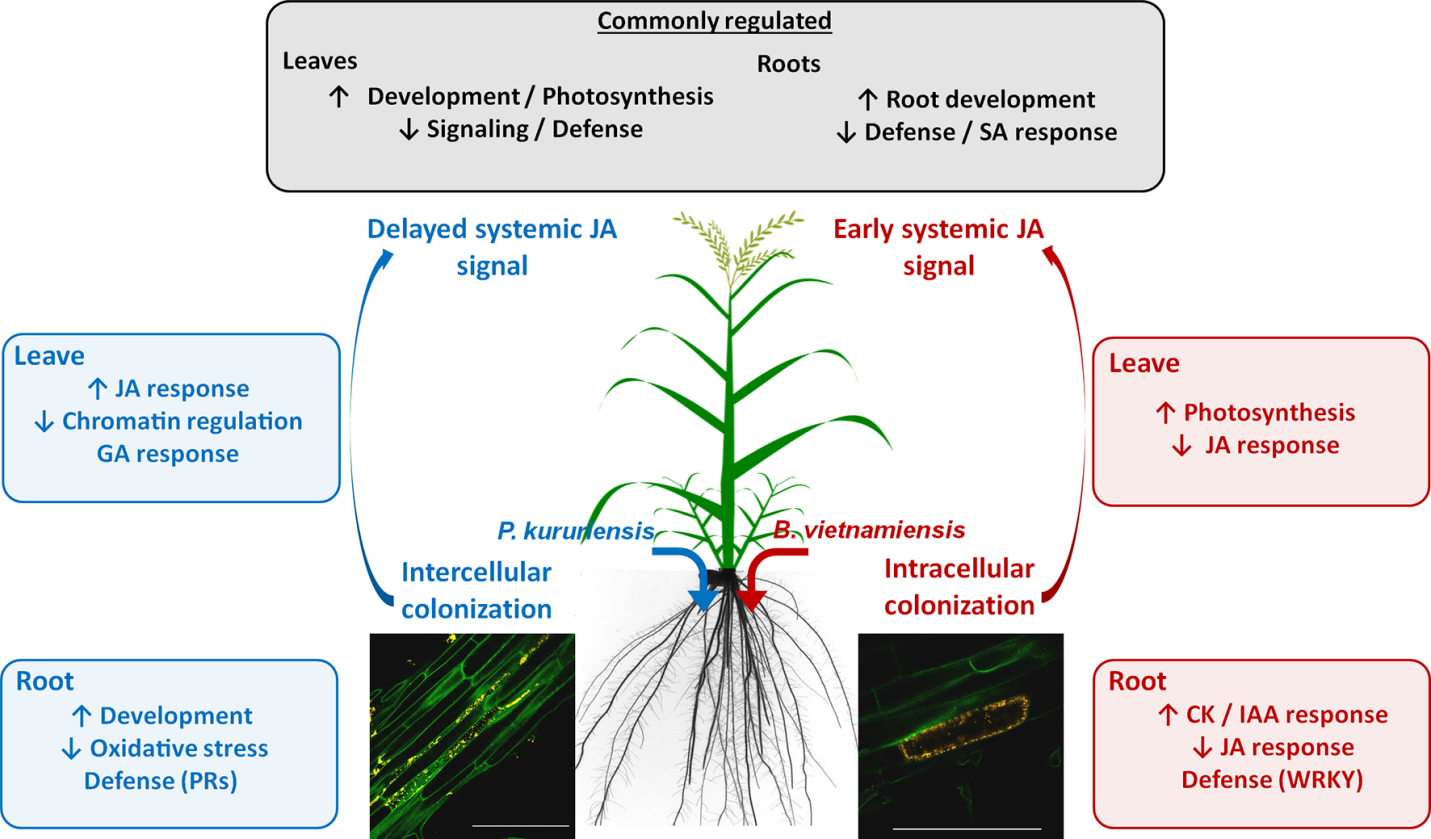
Local and systemic transcriptional regulations of rice in response to *Pk* and *Bv* root colonization. Common responses include the up-regulation of development-related genes and the down-regulation of defense-related genes in both leaves and roots. Strain-specific regulations in roots encompass the down-regulation of different defense-related genes: While *Pk* induced the down-regulation of pathogenesis-related (PR) genes, *Bv* induced the down-regulation of WRKY transcription factors. Root colonization also induced a systemic up-regulation of jasmonic acid-related (JA) genes in leaves, however, not at the same time for each strain: While *Bv* induced this signal at early stages of the interaction, *Pk* induced a transient delayed systemic up-regulation of JA-related genes. *Bv*, *Burkholderia vietnamiensis* TVV75^T^; *Pk*, *Paraburkholderia kururiensis* M130.

The comparison of the interactions between rice and *Pk* and between rice and *Bv* revealed important differences in the process of root colonization and rice transcriptional regulations induced by each strain, which we summarize in **Figure 7**. First, the numerous intracellular colonization of root epidermic cells by *Bv* resembles a pathogen infection compared with the apoplastic colonization observed for *Pk* and other beneficial endophytes (McCully, 2001; Reinhold-Hurek et al., 2007). However, such intracellular colonization by beneficial endophytes has already been observed (Reinhold-Hurek and Hurek, 1998). Also, the specific root response to *Pk* is characterized by the down-regulation of gene coding for chitinase and PR proteins, while *Bv* colonization specifically induced the down-regulation of several *WRKY* genes (**Table 3**). Moreover, *Bv* specifically induced the down-regulation of JA signaling genes (**Supplementary Figure 5**; **Supplementary Table 6**) in addition to the common SA-related down-regulation in roots. Therefore, the strategies to circumvent the immune system of roots appear to be different between the two strains. Finally, the fact that the root inoculation of *Bv* induced the up-regulation of JA-related genes in leaves in only 6 hpi compared with the delayed similar signal in response to *Pk* colonization at 7 dpi also supports the fact that *Bv* induces a response similar to that of pathogens. Indeed, the rice root colonization by *M. oryzae* induced the up-regulation of JA-related genes in leaves at 3 and 4 dpi (Marcel et al., 2010). All these elements support the fact that *Bv* appears to have a much more invasive colonization strategy in terms of both patterns and modulation of the plant immune system. This statement is in accordance with the opportunistic and pathogenic background of the *Burkholderia s.s.* genus (Eberl and Vandamme, 2016). Consequently, it would be of interest to analyze the response of the cultivar *Nipponbare* to a larger diversity of plant-associated *Burkholderia* and *Paraburkholderia* species to investigate if the differences observed between the two strains can be extrapolated to other species of the respective clades.

Another interesting conclusion from this study is the importance of the JA signaling in the interaction with beneficial rhizobacteria. As previously discussed, this major component of plant defense classically associated with the resistance to herbivores and necrotrophic pathogens appears to be involved in a larger diversity of biotic interactions (Thaler, 2004). We have demonstrated that there is a temporal delay in the induction of JA-related genes in leaves following the colonization by *Pk* comparatively to the response to *Bv*. It would be of interest to investigate the role of JA in the establishment of these interactions in terms of colonization level and plant defense status using JA-deficient mutants.

## Data Availability

These sequence data for this study can be found in the EMBL database under accession number PRJEB31936.

## Author Contributions

EK, PC, and LM contributed to the conception and design of the study. EK, AW, IR, CB, and AK performed data collection and analysis. EK wrote the first draft. PC and LM contributed to manuscript revision. All authors read and approved the submitted version.

## Funding

The authors acknowledge the CGIAR Research Program on Rice Agri-food Systems (RICE) and the MIC-CERES Project (FC Project ID 2013-1888; AF Project ID 1301-003, jointly supported by Agropolis Fondation through the “Investissements d’avenir” programme with reference number ANR-10-LABX-0001-01, and Fondazione Cariplo) for funding. EK and AW were supported by a fellowship from the French Ministry of Higher Education, Research and Innovation.

## Conflict of Interest Statement

The authors declare that the research was conducted in the absence of any commercial or financial relationships that could be construed as a potential conflict of interest.
