# Supplementary material for: Monitoring of Rice Transcriptional Responses to Contrasted Colonizing Patterns of Phytobeneficial Burkholderia s.l. Reveals a Temporal Shift in JA Systemic Response
Source: Front Plant Sci. 2019 Sep 24;10:1141. doi: 10.3389/fpls.2019.01141 (PMC6769109; doi:10.3389/fpls.2019.01141)
Supplement: Supplementary file 1 [file Table_1.docx]

| Supplementary Table 1 : Hydroponic medium | | |
| --- | --- | --- |
| **Solutions** | **Salts** | **Final concentration** |
| NH_4_ + Mg | (NH_4_)_2_SO_4_ | 0,5 mM |
|  | MgSO_4_.7H_2_O | 1,6 mM |
| Ca + K | Ca(NO_3_)_2_.4H_2_O | 1,2 mM |
|  | KNO_3_ | 0,7 mM |
| Oligo-éléments | MnSO_4_.H_2_O | 10 µM |
|  | (NH_4_)_6_Mo_7_O_24_.4H_2_O | 0,16 µM |
|  | ZnSO_4_.7H_2_O | 0,7 µM |
|  | CuSO_4_.5H_2_O | 0,8 µM |
|  | H_3_BO_3_ | 22,6 µM |
| FeEDTA | FeSO_4_.7H_2_O | 90 µM |
|  | EDTA | 90 µM |
| KH_2_PO_4_ | KH_2_PO_4_ | 0,4 mM |
| Si | CaSiO3·9H2O | 1,70 mM |
