# Supplementary material for: Monitoring of Rice Transcriptional Responses to Contrasted Colonizing Patterns of Phytobeneficial Burkholderia s.l. Reveals a Temporal Shift in JA Systemic Response
Source: Front Plant Sci. 2019 Sep 24;10:1141. doi: 10.3389/fpls.2019.01141 (PMC6769109; doi:10.3389/fpls.2019.01141)
Supplement: Supplementary file 2 [file Table_2.doc]

| Supplementary Table 2 : Bacterial strains used in this study | | | | |
| --- | --- | --- | --- | --- |
| **Species name** | **Culture medium** | **Strain** | **Transformation** | **Reference** |
| *Escherichia coli* | LB Cm 30 µg/mL | DH5α | plasmid pIN29 *: ori*_pBBR_ Δ*mob*, Cm^r^, DSRed | Vergunst *et al*., 2010 |
| *Paraburkholderia kururiensis* | LB low salt | M130 | Wild strain | Baldani *et al.*, 1997 |
| *Burkholderia vietnamiensis* | LB low salt | TVV75 (= LMG 10929^T^) | Wild strain | Tran Van *et al*., 1996 |
| *Paraburkholderia kururiensis* | LB low salt Cm 200 µg/mL | M130 + pIN29 | pIN29 by electroporation | This study |
| *Burkholderia vietnamiensis* | LB low saltm Cm 200 µg/mL | TVV75 (= LMG 10929^T^) + pIN29 | pIN29 by electroporation | This study |
