# Supplementary material for: Monitoring of Rice Transcriptional Responses to Contrasted Colonizing Patterns of Phytobeneficial Burkholderia s.l. Reveals a Temporal Shift in JA Systemic Response
Source: Front Plant Sci. 2019 Sep 24;10:1141. doi: 10.3389/fpls.2019.01141 (PMC6769109; doi:10.3389/fpls.2019.01141)
Supplement: Supplementary file 3 [file Table_3.doc]

| Supplementary Table 3 : Primers used in this study | | |  |
| --- | --- | --- | --- |
| RAP ID | Gene name | Primers sequence | Reference |
| Os03g0195100 | *ALD1* | AGGCCACAAGAATCTGAGGAAG | This study |
|  |  | TCTGAAGACGAGCAATGTCACA |  |
| Os03g0741100 | *bHLH148* | ACGATTGCGGCTTGTGAAGT | Caldana *et al*., 2007 |
|  |  | TGTCCAGCCTTTGCTTCCA |  |
| Os11g0143300 | *RR9* | TCATGAGGACAGCCCAATTTCTA | Jain *et al*., 2006 |
|  |  | TGCAGTAGTCTGTGATGATCAGGTT |  |
| Os12g0589000 | *RSL9* | GGGCCACGAGGATCTTGATT | This study |
|  |  | CCACAGTTTGCTTGGCCTTC |  |
| Os08g0203400 | *SHR5* | ATCTCTTTGAGTGGGCTTGGAG | This study |
|  |  | CCCTGCTGAATTCCTCAAGTCT |  |
| Os02g0181300 | *WRKY71* | AGCCTGGTGGTGAAAGATGGGTA | Caldana *et al*., 2007 |
|  |  | CATCTGAAGTAGGCTCTTGGGCAG |  |
| Os04g0301500 | *RERJ1* | ATGGAGTCATGCGTTTTGGC | This study |
|  |  | TGGGGTGTCGCAAAAATGAC |  |
| Os03g0402800 | *JAZ6* | TTGATGACTTCCCAGCTGAGAA | Lu *et al*., 2016 |
|  |  | GCGCTGTGGAGGAACTCTTG |  |
| Os03g0181100 | *JAZ10* | TCTTCCCACCCCGTCAAAT | Zong *et al*., 2016 |
|  |  | CCTCGCTGGTGCTTTGCT |  |
| Os10g0392400 | *JAZ12* | TGCCGATCGCGAGGAA | Hou *et al*., 2009 |
|  |  | GGTTCGCTCGTTGTCGTGAT |  |
| Os03g0767000 | *AOS1* | TTCCTCCGATACGACTCCTTC | This study |
|  |  | AGGTGACGGTGACAGATGAG |  |
