# Supplementary material for: Monitoring of Rice Transcriptional Responses to Contrasted Colonizing Patterns of Phytobeneficial Burkholderia s.l. Reveals a Temporal Shift in JA Systemic Response
Source: Front Plant Sci. 2019 Sep 24;10:1141. doi: 10.3389/fpls.2019.01141 (PMC6769109; doi:10.3389/fpls.2019.01141)
Supplement: Supplementary file 9 [file Table_9.doc]

| Supplementary Table 9 : Genes used to confirm RNAseq and their function | | | | | |
| --- | --- | --- | --- | --- | --- |
| **RAP-ID** | **Gene name** | **Organ** | **Regulation according to RNASeq** | **Function** | **Reference** |
| Os03g0195100 | *ALD1* | Leaves | Down-regulated by *B. vietnamiensis* | Systemic resistance | Jung *et al.*, 2016 |
| Os03g0741100 | *bHLH148* | Roots | Down-regulated by *B. vietnamiensis* | JA responsive | Seo *et al.*, 2011 |
| Os11g0143300 | *RR9* | Leaves | Down-regulated by *B. vietnamiensis* | CK signaling | Ito and Kurata, 2006 |
| Os12g0589000 | *RSL9* | Roots | Commonly up-regulated by both strains | Root hair formation | Min Kim *et al.*, 2017 |
| Os08g0203400 | *SHR5* | Roots | Down-regulated by *P. kururiensis* | Homolog of sugarcane LRR-RLK involved in plant-N2 fixing endophytic association | Vinagre *et al.*, 2006 |
| Os02g0181300 | *WRKY71* | Leaves | Up-regulated by *P. kururiensis* | Disease resistance | Liu *et al.*, 2007 |
