# Supplementary material for: Monitoring of Rice Transcriptional Responses to Contrasted Colonizing Patterns of Phytobeneficial Burkholderia s.l. Reveals a Temporal Shift in JA Systemic Response
Source: Front Plant Sci. 2019 Sep 24;10:1141. doi: 10.3389/fpls.2019.01141 (PMC6769109; doi:10.3389/fpls.2019.01141)
Supplement: Supplementary file 10 [file Table_10.doc]

| Supplementary Table 10: Expression profiles of the JA network in leaves. Values are log2FoldChange of expression compared to non-inoculated plants measured by qRT-PCR. | | | | | | | | |
| --- | --- | --- | --- | --- | --- | --- | --- | --- |
|  | 6 hpi | | 1 dpi | | 7 dpi | | 14 dpi | |
|  | *Pk* | *Bv* | *Pk* | *Bv* | *Pk* | *Bv* | *Pk* | *Bv* |
| *ATL15* | -0,80 | 1,05 | -0,28 | -0,69 | 4,60 | -0,04 | -1,52 | -1,86 |
| *RERJ1* | 0,89 | 1,93 | -0,22 | 1,49 | 0,40 | 0,49 | -0,36 | -1,24 |
| *AOS1* | 0,56 | 0,36 | -2,05 | -1,66 | 1,71 | 0,67 | -1,54 | -1,92 |
| *JAZ6* | -0,16 | 1,04 | -2,33 | -1,19 | 1,53 | -0,82 | -0,28 | -0,66 |
| *JAZ10* | 0,12 | 1,68 | -1,20 | 0,60 | 2,52 | -0,64 | -1,79 | -1,40 |
| *JAZ12* | 2,26 | 1,92 | -3,19 | -0,54 | 1,61 | -0,42 | -2,46 | -1,24 |
