# Supplementary figures and images for: Monitoring of Rice Transcriptional Responses to Contrasted Colonizing Patterns of Phytobeneficial Burkholderia s.l. Reveals a Temporal Shift in JA Systemic Response
Source: Front Plant Sci. 2019 Sep 24;10:1141. doi: 10.3389/fpls.2019.01141 (PMC6769109; doi:10.3389/fpls.2019.01141)

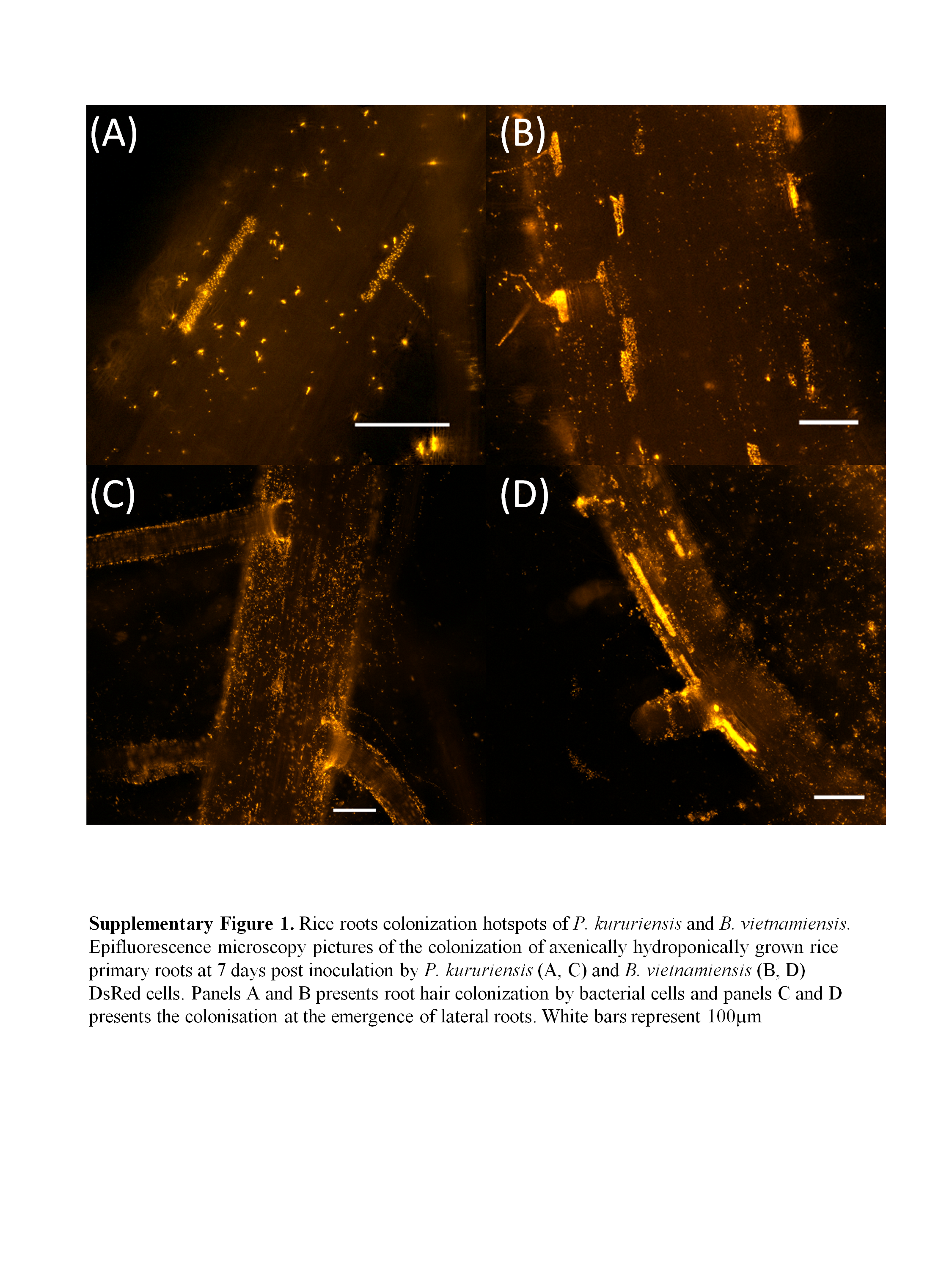

Supplement: Supplementary file 11 [file Image_1.tif]

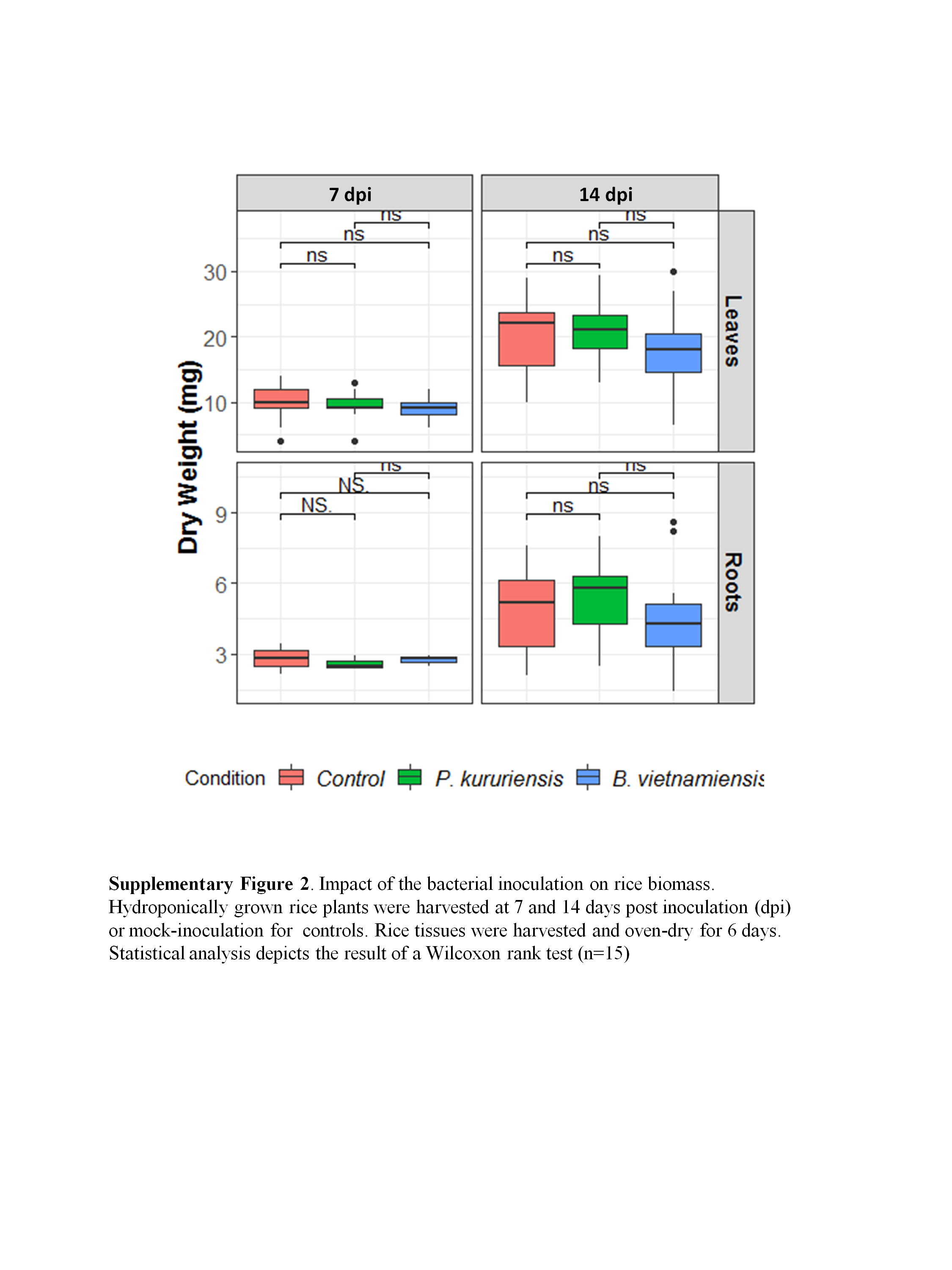

Supplement: Supplementary file 12 [file Image_2.tif]

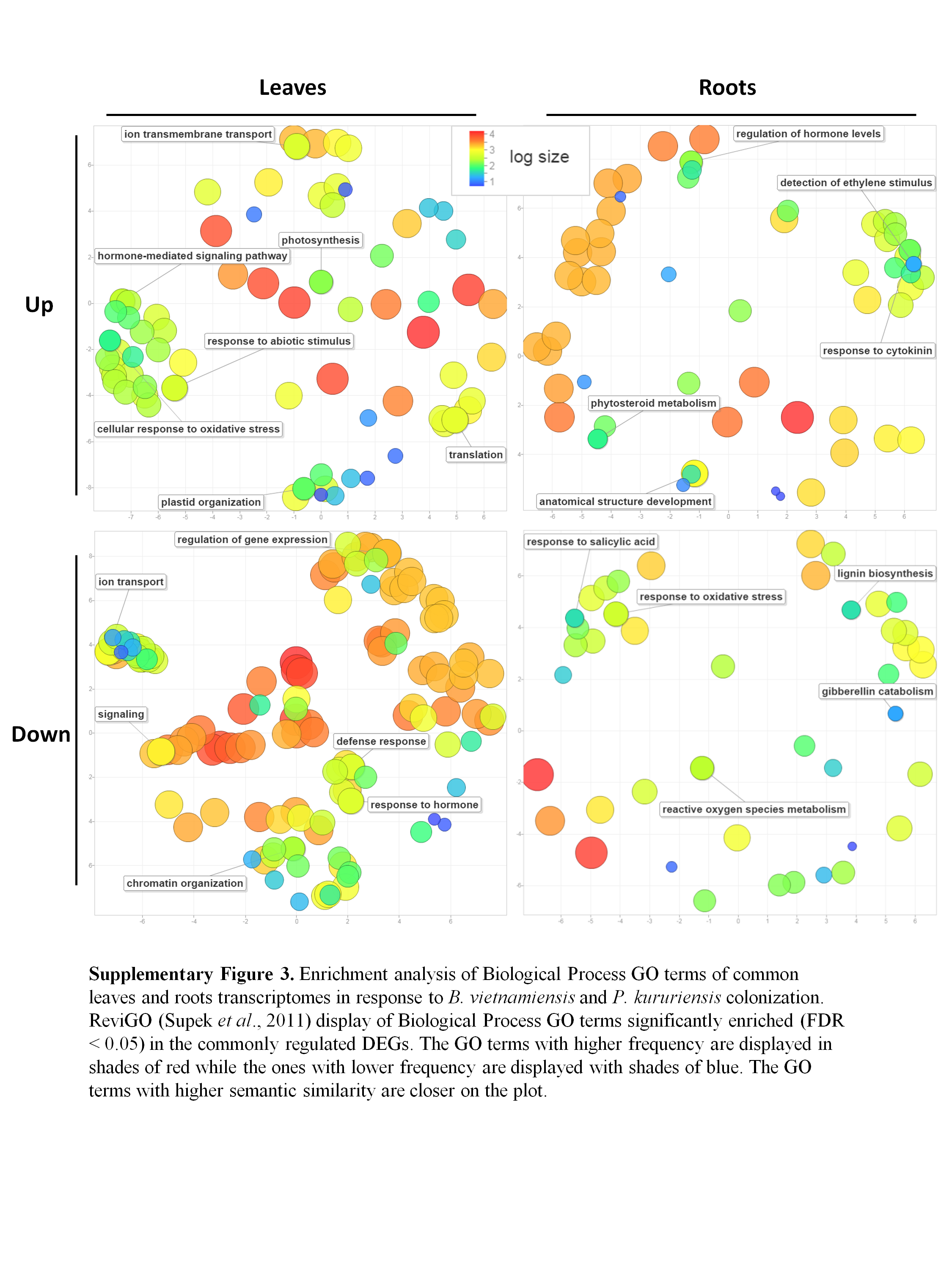

Supplement: Supplementary file 13 [file Image_3.tif]

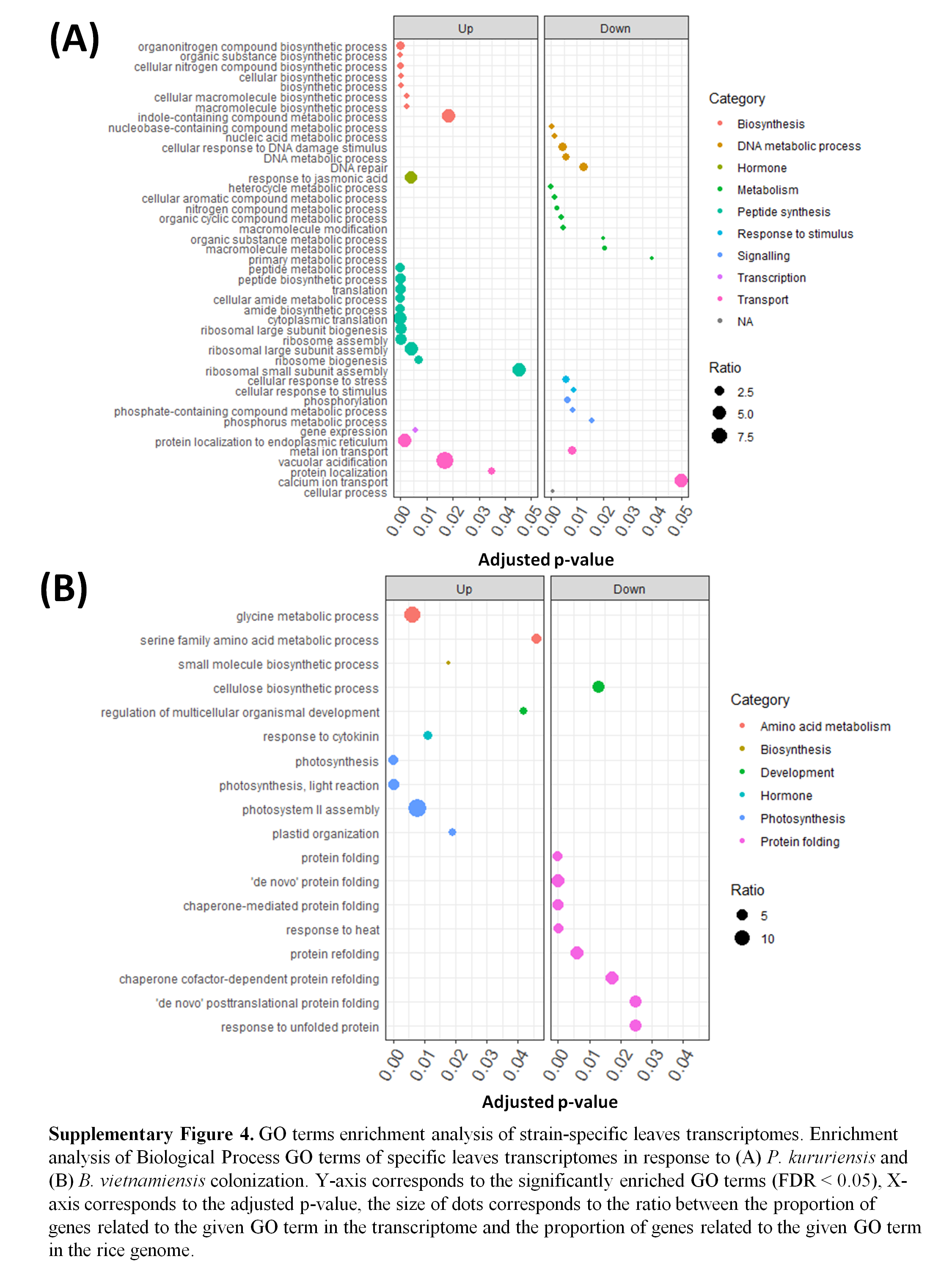

Supplement: Supplementary file 14 [file Image_4.tif]

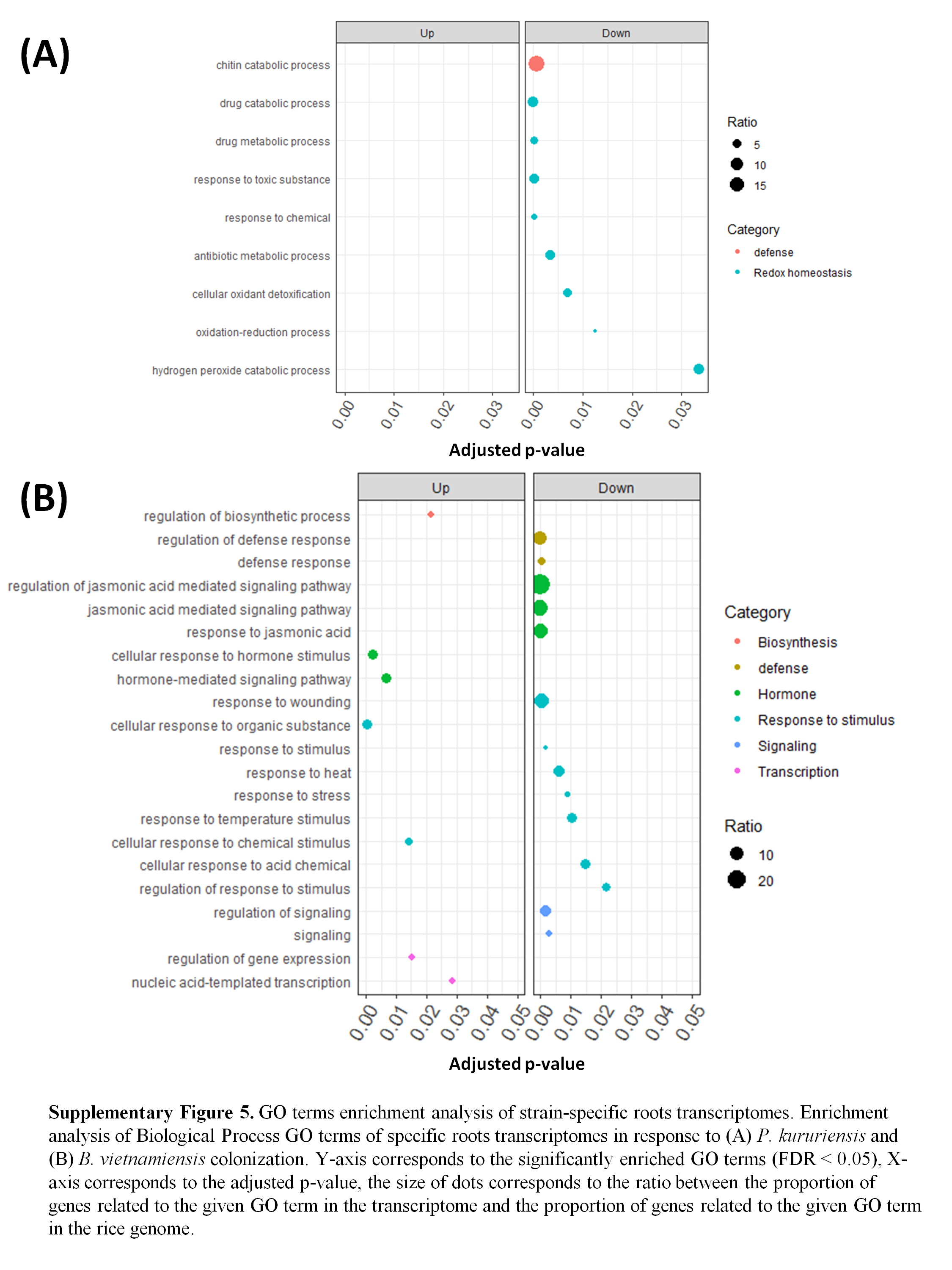

Supplement: Supplementary file 15 [file Image_5.tif]
