# Supplementary material for: Monitoring of Rice Transcriptional Responses to Contrasted Colonizing Patterns of Phytobeneficial Burkholderia s.l. Reveals a Temporal Shift in JA Systemic Response
Source: Front Plant Sci. 2019 Sep 24;10:1141. doi: 10.3389/fpls.2019.01141 (PMC6769109; doi:10.3389/fpls.2019.01141)
Supplement: Supplementary file 16 [file DataSheet_1.doc]

**Supplementary Material References**

Baldani, V. L. D., Oliveira, E., Balota, E., Baldani, J. I., Kirchhof, G., and Dobereiner, J. (1997). *Burkholderia brasilensis sp. nov.*, uma nova especie de bacteria diazotrofica endofitica. *An. Acad. Bras. Cienc.* 69, 116.

Caldana, C., Scheible, W.-R., Mueller-Roeber, B., and Ruzicic, S. (2007). A quantitative RT-PCR platform for high-throughput expression profiling of 2500 rice transcription factors. *Plant Methods* 3, 7. doi:10.1186/1746-4811-3-7.

Hou, X., Xie, K., Yao, J., Qi, Z., and Xiong, L. (2009). A homolog of human ski-interacting protein in rice positively regulates cell viability and stress tolerance. *Proc. Natl. Acad. Sci.* 106, 6410–6415. doi:10.1073/pnas.0901940106.

Ito, Y., and Kurata, N. (2006). Identification and characterization of cytokinin-signalling gene families in rice. *Gene* 382, 57–65. doi:10.1016/J.GENE.2006.06.020.

Jain, M., Tyagi, A., and Khurana, J. (2006). Molecular characterization and differential expression of cytokinin-responsive type-A response regulators in rice ( Oryza sativa ). *BMC Plant Biol.* 6, 1. doi:10.1186/1471-2229-6-1.

Lu, Y. C., Feng, S. J., Zhang, J. J., Luo, F., Zhang, S., and Yang, H. (2016). Genome-wide identification of DNA methylation provides insights into the association of gene expression in rice exposed to pesticide atrazine. *Sci. Rep.* 6, 18985. doi:10.1038/srep18985.

Min Kim, C., Han, C., and Dolan, L. (2017). RSL class I genes positively regulate root hair development in Oryza sativa. *New Phytol.* 213, 314–323. doi:10.1111/nph.14160.

Seo, J.-S., Joo, J., Kim, M.-J., Kim, Y.-K., Nahm, B. H., Song, S. I., et al. (2011). OsbHLH148, a basic helix-loop-helix protein, interacts with OsJAZ proteins in a jasmonate signaling pathway leading to drought tolerance in rice. *Plant J.* 65, 907–921. doi:10.1111/j.1365-313X.2010.04477.x.

Supek, F., Bošnjak, M., Škunca, N., and Šmuc, T. (2011). REVIGO Summarizes and Visualizes Long Lists of Gene Ontology Terms. *PLoS One* 6, e21800. doi:10.1371/journal.pone.0021800.

Trân Van, V., Berge, O., Balandreau, J., Ngô Ké, S., and Heulin, T. (1996). Isolement et activité nitrogénasique de *Burkholderia vietnamiensis*, bactérie fixatrice d’azote associée au riz (*Oryza sativa L*) cultivé sur un sol sulfaté du Viêt-nam. *Agronomie* 16, 479–491. doi:10.1051/agro:19960802.

Vinagre, F., Vargas, C., Schwarcz, K., Cavalcante, J., Nogueira, E. M., Baldani, J. I., et al. (2006). SHR5: a novel plant receptor kinase involved in plant–N2-fixing endophytic bacteria association. *J. Exp. Bot.* 57, 559–569. doi:10.1093/jxb/erj041.

Wang, M., Wei, P., Cao, M., Zhu, L., and Lu, Y. (2016). First Report of Rice Seedling Blight Caused by *Burkholderia plantarii* in North and Southeast China. *Plant Dis.* 100, 645. doi:10.1094/PDIS-07-15-0765-PDN.

Zong, W., Tang, N., Yang, J., Peng, L., Ma, S., Xu, Y., et al. (2016). Feedback regulation of ABA signaling and biosynthesis by a bZIP transcription factor targets drought resistance related genes. *Plant Physiol.* 171, pp.00469.2016. doi:10.1104/pp.16.00469.
